# Analysis of the interaction of extracellular matrix and phenotype of bladder cancer cells

**DOI:** 10.1186/1471-2407-6-12

**Published:** 2006-01-13

**Authors:** Mikhail G Dozmorov, Kimberly D Kyker, Ricardo Saban, Nicholas Knowlton, Igor Dozmorov, Michael B Centola, Robert E Hurst

**Affiliations:** 1 Department of Urology, Oklahoma University Health Sciences Centre, Oklahoma City, OK 73104, USA; 2 Department of Biochemistry and Molecular Biology, Oklahoma University Health Sciences Centre, Oklahoma City, OK 73104, USA; 3 Department of Occupational and Environmental Health, Oklahoma University Health Sciences Centre, Oklahoma City, OK 73104, USA; 4 Microarray Core Facility, Oklahoma Medical Research Foundation, Oklahoma City, OK 73104, USA; 5 Department of Physiology, Oklahoma University Health Sciences Centre, Oklahoma City, OK 73104, USA

## Abstract

**Background:**

The extracellular matrix has a major effect upon the malignant properties of bladder cancer cells both *in vitro *in 3-dimensional culture and *in vivo*. Comparing gene expression of several bladder cancer cells lines grown under permissive and suppressive conditions in 3-dimensional growth on cancer-derived and normal-derived basement membrane gels respectively and on plastic in conventional tissue culture provides a model system for investigating the interaction of malignancy and extracellular matrix. Understanding how the extracellular matrix affects the phenotype of bladder cancer cells may provide important clues to identify new markers or targets for therapy.

**Methods:**

Five bladder cancer cell lines and one immortalized, but non-tumorigenic, urothelial line were grown on Matrigel, a cancer-derived ECM, on SISgel, a normal-derived ECM, and on plastic, where the only ECM is derived from the cells themselves. The transcriptomes were analyzed on an array of 1186 well-annotated cancer derived cDNAs containing most of the major pathways for malignancy. Hypervariable genes expressing more variability across cell lines than a set expressing technical variability were analyzed further. Expression values were clustered, and to identify genes most likely to represent biological factors, statistically over-represented ontologies and transcriptional regulatory elements were identified.

**Results:**

Approximately 400 of the 1186 total genes were expressed 2 SD above background. Approximately 100 genes were hypervariable in cells grown on each ECM, but the pattern was different in each case. A core of 20 were identified as hypervariable under all 3 growth conditions, and 33 were hypervariable on both SISgel and Matrigel, but not on plastic. Clustering of the hypervariable genes showed very different patterns for the same 6 cell types on the different ECM. Even when loss of cell cycle regulation was identified, different genes were involved, depending on the ECM. Under the most permissive conditions of growth where the malignant phenotype was fully expressed, activation of AKT was noted. TGFβ1 signaling played a major role in the response of bladder cancer cells to ECM. Identification of TREs on genes that clustered together suggested some clustering was driven by specific transcription factors.

**Conclusion:**

The extracellular matrix on which cancer cells are grown has a major effect on gene expression. A core of 20 malignancy-related genes were not affected by matrix, and 33 were differentially expressed on 3-dimensional culture as opposed to plastic. Other than these genes, the patterns of expression were very different in cells grown on SISgel than on Matrigel or even plastic, supporting the hypothesis that growth of bladder cancer cells on normal matrix suppresses some malignant functions. Unique underlying regulatory networks were driving gene expression and could be identified by the approach outlined here.

## Background

Bladder cancer has the fourth highest incidence in men and seventh in women in the US [1]. Superficial papillary bladder cancers, though they may grow very large, do not invade and generally can be managed by excision. The more aggressive invasive bladder cancers that develop *de novo *have a five-year survival rate of 5% or less if metastasis has occurred [2,3]. Although 85% of bladder cancers first appear as superficial papillary carcinomas, up to two-thirds of patients experience recurrence, with approximately 15–25% progressing to higher grade tumors [2,3]. Approximately half of the deaths from bladder cancer result from progression. The extracellular matrix plays a crucial role in the development and evolution of bladder cancers [4-6]. The finding that specific genetic alterations frequently associated with bladder cancer are detectable in histologically normal urothelium of patients with bladder cancer [7] suggests that the malignant phenotype can be suppressed *in vivo*. This observation strongly suggests that understanding how the extracellular matrix modulates the phenotype of bladder cancer cells is highly relevant to understanding the processes of recurrence and progression.

Models for investigating interactions between the extracellular matrix (ECM) and bladder cancer cells using 3-dimensional culture have shown the ECM plays a crucial role in modulating the phenotype of bladder cancer cells [4,5,8-10]. Previous studies in our laboratory show suppression of the malignant phenotype when cells are cultured on a normal ECM [9,10]. On Matrigel, which is prepared from a cancer-modified ECM, bladder cancer cells recapitulate their *in vivo *phenotype. In contrast, when grown on SISgel, which is prepared from normal basement membrane ECM, the tumorigenic cell lines lose their invasiveness or ability to form papillary structures. Instead, they form either multiple or single layers resembling normal urothelium. By systematically varying both malignancy in the form of the cell line and the ECM on which they are grown, the interaction between these biological variables can be examined at the level of gene transcription. In this study, expression of 1186 well-annotated cancer-associated genes was measured on a cDNA array. The study design involved 6 cell lines varying from non-malignant to highly malignant grown on two ECM preparations and on plastic. Because practical considerations limited the number of replicates to 2, the dataset was inherently "noisy." We therefore developed statistically valid, novel approaches to data analysis that can identify biologically relevant patterns in a noisy dataset. Application of these methods for the given dataset yielded clear distinction in gene expression between different cell lines and/or matrixes.

## Methods

### Cell culture

SV-HUC-1, TCCSUP, RT4 and J82 cells were obtained from the American Type Culture Collection, Bethesda, MD, which provided information allowing the cells to be ranked by malignancy of the tumor of origin. The 253 J and 253 JB-V cells were provided by Dr. Colin Dinney [11]. The former is derived from a metastatic tumor, while the latter is a highly metastatic variant cloned in Dr. Dinney's laboratory after 5 passages of 253 J cells in the bladder walls of nude mice. Although invasive and metastatic, the tumor morphology is papillary. The ranking according to malignancy from lowest to highest is: SV-HUC-1 (non-malignant but immortalized), RT4 (low grade), 253 J (high grade) 253 JB-V (high grade), J82 (high grade), TCCSUP (high grade). Excepting the non-malignant SV-HUC-1 cells and possibly the TCCSUP cells (see below), all the cancer cell lines are transitional cell carcinomas (TCC). Details of cell culture on Matrigel and SISgel have been reported previously [9,10]. Culture on both matrices plus plastic in which the proliferation rate is approximately the same in all 3 has been described previously [12].

### Array protocol

RNA was isolated from the cells growing in 3 dimensional culture using the RNeasy kit (Qiagen) by adding 300 μl lysis buffer to the culture well and pipeting up and down to lyse the cells and dissolve the gel. The RNA was isolated from the lysate using a QIAshredder spin column to complete homogenization followed by proteinase K digestion, washing, DNase 1 treatment and elution from RNeasy spin column. Yield of total RNA and the purity were assessed by spectrophotometry. The yield was approximately 2.4 – 6 μg RNA per culture in 30 μl. First strand synthesis was carried out at 42°C for 1 h with reagents supplied by Clontech as part of the SMART II kit, except that Superscript II (Invitrogen) reverse transcriptase was substituted. The SMART II oligonucleotide was included to capture full length cDNA at the 5'end and to permit a proportional 2,000 – 5,000 fold amplification [13]. Labeled probe was produced from an aliquot of the amplified probe with Klenow fragment and 32P-labeled nucleotide. After probe purification, 15 × 106 cpm of probe was hybridized in duplicate to the Clontech Human Cancer 1.2 array on nylon following the protocol provided by the company. The Clontech Human Cancer 1.2 array is a focused array consisting of 1186 well-annotated cancer-associated genes. The results were visualized by exposing a phosphoimager screen overnight and then reading with the Packard Cyclone phosphoimager and Optiquant software.

### Data normalization

Data were normalized as described in detail elsewhere [14]. In brief, the normalization method relies on a number of low expression genes to provide an estimate of non-specific binding. This information is then used to perform a Z transformation on the data. Once normalized, the data are "unbiased" through robust linear regression to allow comparisons across different arrays. Fitted data are then used to find a homoscedastic group of gene variances that will be used as an internal standard of measurement noise [15]. Expressed genes (expression levels 2 standard deviations above the mean of the background) were noted, anti-log transformed, and used for subsequent analysis. This filtering step minimizes false positives, though at the cost of the low-expression genes, which are measured with low precision anyway.

### Hypervariable gene identification

The logic of this step is that the variability within a dataset consists of both experimental error and variability introduced by the biological variables of interest. Moreover, while genes that do not vary significantly may be representing ongoing physiologic processes, they are unlikely to be correlated with malignancy or the effect of ECM since they do not change with either of those variables. Dozmorov, et al. [14] presented a method to identify the subset of genes that most likely represent experimental variability. Those genes with statistically significant variability greater than this subset represent the hypervariable genes that are most likely to represent the action of biological variables. Thus, in this study any gene that showed variable expression across cell lines exceeding the estimate of technical variability would be considered hypervariable. In order to reduce noise in the dataset and to insure adequate statistical power, the list was filtered to remove genes that were not expressed 2 SD above background in at least 4 of 6 cell lines for growth on each ECM condition. This filtering reduced the 1186 genes to roughly 400 for each matrix, with about 100 being identified as hypervariable.

### Correlational clustering

The expression values of the hypervariable genes were clustered using Gene Cluster from M. B. Eisen [16]. The data were log-transformed, arrays and genes were median-centered and the transformed dataset was hierarchically clustered using uncentered correlational clustering. The results were displayed with TreeView, also from M. B. Eisen.

### Identification of significantly over-represented gene ontologies

The annotations of the gene lists and their individual ontologies were first updated using the publicly available on-line tool DAVID EASE [17]. The null hypothesis that the clusters identified arose by chance was tested by determining whether any gene ontologies were over-represented above chance using the Gene Ontology Tree Machine (GOTM) [18,19].

### Pathway analysis

Biologically relevant networks were assembled from identified clusters and groups of common genes by using Ingenuity Pathways Analysis (IPA). This web-delivered application [20] enables the visualization and analysis of direct and indirect interactions among genes. Selected data sets were uploaded as Excel files containing GenBank Accession numbers for genes of interest. Each gene identifier was mapped to its corresponding gene object in the Ingenuity Pathways Knowledge Base. Genes were not weighted by expression levels, and biological networks were built on this assumption.

### Transcriptional regulatory element (TRE) analysis

One strategy for analyzing the significance of gene clusters identified by correlational clustering is based upon the assumption that co-expressed genes are likely to share common regulatory motifs [21]. This analysis was performed using the web-based program PAINT [22,23]. The results depend upon the reference chosen thereby reflecting Bayesian probability. If the entire genome is selected as the reference, the results will emphasize TREs common to bladder cells and would therefore identify tissue-specific TREs. Alternately, if the list of all genes expressed on a given matrix is selected as the reference, then the results should be enriched in those TREs responsible for the clustering rather than tissue-specific promoters. If the reference is the list of hypervariable genes, then significance of the partitioning of the hypervariable genes by clusters and TREs is identified. The PAINT analysis was performed against each of the indicated references, but only the reference against all hypervariable genes was reported.

## Results

### Identification of hypervariable genes

**Figure 1:**
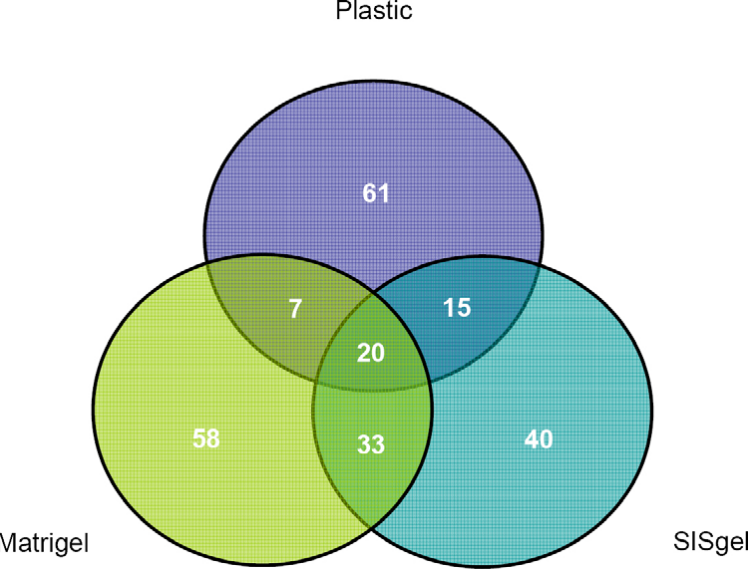
***Venn diagram of hypervariable genes expressed on different matrixes***. Each circle contains number of hypervariable genes unique and/or common for different matrixes. The size of circles does not represent relative number of genes in each group.

Cells grown on plastic expressed 422/1186 genes in at least 4/6 cell lines. On SISgel and Matrigel the corresponding numbers were 411 and 409 respectively. Overall, 371 genes were expressed in at least 12/18 conditions (6 cell lines grown under 3 conditions). The lists were further filtered by identifying the set of hypervariable genes. This yielded lists of approximately 100 genes from each set of experiments on an individual matrix. The results are expressed by a Venn diagram (Figure 1). The full gene lists and descriptions are available [see Additional file 1]. Figure 1 shows roughly half of each set of hypervariable genes was expressed uniquely on one ECM. Only 20 genes were expressed in common on all three ECMs (Table 1), and 33 genes were showed hypervariable expression on both SISgel and Matrigel (Table 2), whereas 15 and 7 were hypervariable on plastic and either SISgel and Matrigel, respectively. Full ontologic descriptions of these 20 and 33 genes are presented [see Additional file 2, Additional file 3].

**Table 1:** Hypervariable genes commonly expressed between Matrigel, Plastic and SISgel

***GENBANK***	***SYMBOL***	***GENENAME***
U13696	PMS2	PMS2 postmeiotic segregation increased 2 (S. cerevisiae)
M68520	CDK2	cyclin-dependent kinase 2
X80692	MAPK6	mitogen-activated protein kinase 6
X76104	DAPK1	death-associated protein kinase 1
S85655	PHB	prohibitin
AF000546	P2RY5	purinergic receptor P2Y, G-protein coupled, 5
M97934	STAT2	signal transducer and activator of transcription 2, 113 kDa
X02851	IL1A	interleukin 1, alpha
X86779	FASTK	FAST kinase
U56390	CASP9	caspase 9, apoptosis-related cysteine protease
U60520	CASP8	caspase 8, apoptosis-related cysteine protease
X57766	MMP11	matrix metalloproteinase 11 (stromelysin 3)
M55172	AGC1	aggrecan 1 (chondroitin sulfate proteoglycan 1, large aggregating proteoglycan, antigen identified by monoclonal antibody A0122)
X03168	VTN	vitronectin (serum spreading factor, somatomedin B, complement S-protein)
U65410	MAD2L1	MAD2 mitotic arrest deficient-like 1 (yeast)
X06374	PDGFA	platelet-derived growth factor alpha polypeptide
L27943	CDA	cytidine deaminase
X03124	TIMP1	tissue inhibitor of metalloproteinase 1 (erythroid potentiating activity, collagenase inhibitor)
A14844	IL2	interleukin 2
M26326	KRT18	keratin 18

**Table 2:** Hypervariable genes commonly expressed between Matrigel and SISgel

***GENBANK***	***SYMBOL***	***GENENAME***
U13695	PMS1	PMS1 postmeiotic segregation increased 1 (S. cerevisiae)
M63167	AKT1	v-akt murine thymoma viral oncogene homolog 1
M11810	OAS1	2',5'-oligoadenylate synthetase 1, 40/46 kDa
X05360	CDC2	cell division cycle 2, G1 to S and G2 to M
M32865	G22P1	thyroid autoantigen 70 kDa (Ku antigen)
M57627	IL10	interleukin 10
S74678	HNRPK	heterogeneous nuclear ribonucleoprotein K
U69127	FUBP3	far upstream element (FUSE) binding protein 3
D17516	ADCYAP1R1	adenylate cyclase activating polypeptide 1 (pituitary) receptor type I
U22398	CDKN1C	cyclin-dependent kinase inhibitor 1C (p57, Kip2)
L25081	ARHC	ras homolog gene family, member C
M35416	RALB	v-ral simian leukemia viral oncogene homolog B (ras related; GTP binding protein)
M11886	HLA-C	major histocompatibility complex, class I, C
X74295	MGC17301	hypothetical protein MGC17301
X02812	TGFB1	transforming growth factor, beta 1 (Camurati-Engelmann disease)
M18082	SERPINB2	serine (or cysteine) proteinase inhibitor, clade B (ovalbumin), member 2
K02770	IL1B	interleukin 1, beta
U60519	CASP10	caspase 10, apoptosis-related cysteine protease
D13866	CTNNA1	catenin (cadherin-associated protein), alpha 1, 102 kDa
U09579	CDKN1A	cyclin-dependent kinase inhibitor 1A (p21, Cip1)
U78095	SPINT2	serine protease inhibitor, Kunitz type, 2
U53446	DAB2	disabled homolog 2, mitogen-responsive phosphoprotein (Drosophila)
X02811	PDGFB	platelet-derived growth factor beta polypeptide (simian sarcoma viral (v-sis) oncogene homolog)
X91940	WNT8B	wingless-type MMTV integration site family, member 8B
X74295	ITGA7	integrin, alpha 7
M30938	XRCC5	X-ray repair complementing defective repair in Chinese hamster cells 5 (double-strand-break rejoining; Ku autoantigen, 80 kDa)
L07515	CBX5	chromobox homolog 5 (HP1 alpha homolog, Drosophila)
M34225	KRT8	keratin 8
J05593	TIMP2	tissue inhibitor of metalloproteinase 2
D83597	LY64	lymphocyte antigen 64 homolog, radioprotective 105 kDa (mouse)
M82882	ELF1	E74-like factor 1 (ets domain transcription factor)
J00209	IFNA10	interferon, alpha 10
U09825	TRIM26	tripartite motif-containing 26

### Clustering of hypervariable genes

**Figure 2:**
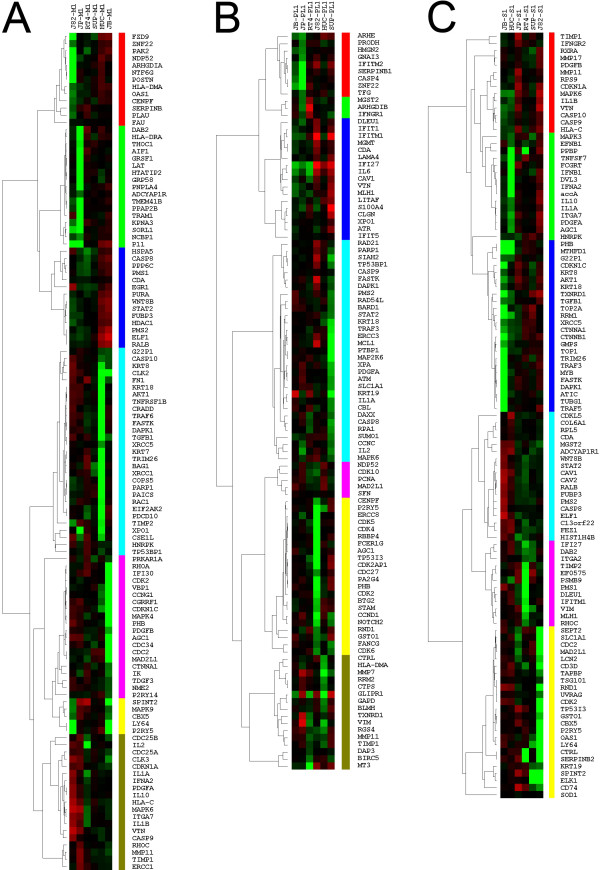
***Hierarchical clustering of hypervariable genes across different cell lines on different matrixes***. Hierarchical clustering of genes up- (red color) or downregulated (green color) on the given 6 cell lines. **A**, **B **and **C **show clustering of genes expressed on Matrigel, Plastic and SISgel, respectively. Colored bars outline individual clusters used for subsequent analysis.

Hierarchical clustering by correlation of gene expression is presented in Figure 2. Gene expression from cells grown on Matrigel, where the bladder cancer cells are capable of fully expressing their malignant phenotype, clustered into seven main correlational clusters (M1–M7) (Figure 2A). The correlations in expression in some of the clusters, such as M2 and M4, are very high (>0.8). The clustering tended to be driven by expression in individual cell types rather than groups, such as cells derived from low-grade tumors. The driving signature (the cell line that drives organization of a cluster), and the over-represented ontologies are summarized in Table 3. The full statistical information as well as gene names associated with individual ontologies are listed [see Additional file 4].

**Table 3:** Driving signatures and ontologies of gene clusters on different matrixes

***Cluster***	***Driving signature***	***Ontology***
Matrigel_1	J82 underexpressed	negative regulation of biological process
Matrigel_2	JP underexpressed	mRNA processing
Matrigel_3	HUC & JB overexpressed	mismatch repair; transcription factor activity
Matrigel_4	HUC underexpressed	anti-apoptosis; induction of apoptosis by extracellular signals; protein binding; protein serine/threonine kinase activity
Matrigel_5	JB underexpressed	regulation of cyclin dependent protein kinase activity; mitotic control; negative regulation of cell proliferation
Matrigel_6	JB & J82 underexpressed	
Matrigel_7	J82 & JP overexpressed	regulation of cyclin dependent protein kinase activity; anti-apoptosis; negative regulation of cell proliferation; positive regulation of cell proliferation; extracellular space
Plastic_1	JP underexpressed	induction of apoptosis
Plastic_2	JP underexpressed RT4 overexpressed	immune response
Plastic_3	TCCSUP & J82 overexpressed	negative regulation of cell cycle
Plastic_4	TCCSUP underexpressed	protein kinase cascade; meiotic recombination; cell cycle arrest; anti-apoptosis; induction of apoptosis by extracellular signals; protein ubiquitination; nucleotide-excision repair; protein binding; nucleus
Plastic_5		mitotic cell cycle; chromosome
Plastic_6	J82 underexpressed	cell cycle arrest; nucleus
Plastic_7	HUC underexpressed	negative regulation of cellular process; antioxidant activity; extracellular matrix
SISgel_1	J82 & JP overexpressed	cell cycle; induction of apoptosis; extracellular matrix
SISgel_2	HUC underexpressed J82 overexpressed	regulation of cell cycle; negative regulation of cell proliferation; inflammatory response
SISgel_3	JB underexpressed	induction of apoptosis by extracellular signals; DNA replication; purine nucleotide biosynthesis; ligase activity, forming carbon-nitrogen bonds; cytoskeleton
SISgel_4	JB overexpressed	
SISgel_5	RT4 underexpressed	negative regulation of cell cycle
SISgel_6	J82 underexpressed	cell division; protein complex assembly

### Analysis of biological pathways

Ingenuity Pathways Analysis identified specific biological pathways behind each given cluster on different ECMs. Cluster M4, containing 29 genes underexpressed in non-malignant HUC cells, showed two non-overlapping networks of 16 and 10 genes, respectively. The first pathway involved TGFβ1 signaling. The second pathway involved p53, IFNG and Fos-responsive genes. The details of the genes identified and their functions are listed in Table 4. The pathways showing the interconnections among the genes are shown [see Additional file 6]. Cluster P7, which consists of 16 genes underexpressed in HUC cells, showed a network of 12 genes involving TGFβ1 and p53 as central players (Table 4).

**Table 4:** Genes and Functions in Ingenuity Pathways

***Cluster/Network***	***Genes***	***Top Functions***
Matrigel_4/1	AKT1, CLK2, CSE1L, DAPK1, FN1, G22P1, KRT7, KRT8, KRT18, PARP1, RAC1, TGFB1, TIMP2, TNFRSF1B, XRCC1, XRCC5	DNA replication, Recombination and Repair; Cell Death; Neurological Disease
Matrigel_4/2	BAG1, CASP10, COPS5, CRADD, EIF2AK2, FASTIK, HNRPK, TP53BP1, TRAF6, XPO1	Cell Cycle; Connective Tissue Disorders; Inflammatory Disease
Plastic_7/1	BIRC5, CTPS, DAP3, GADP, GLIPR1, HLA-DMA, MMP7, MMP11, MT3, RRM2, TIMP1, TXNRD1	Cancer; Cell Death; Respiratory Disease
Mat-Pla-SIS Common Genes/1	AGC1, CASP8, CASP9, CDK2, DAPK1, FASTK, IL2, IL1A, KRT18, MAD2L1, MAPK6, MMP11, PDGFA, PHB, PMS2, STAT2, TIMP1, VNT	Cell Death; Cancer; Cell Cycle
Mat-SIS Common Genes/1	ADCYAP1R1, AKT1, CDC2, CDKN1A, CDKN1C, HLA-C, IL10, IL1B, ITGA7, KRT8, PDGFB, PMS1, SERPINB2, SPINT2, TGFB1	Cancer; Cell Morphology; Cell Cycle
Mat-SIS Common Genes/2	CASP10, CBX5, CTNN1, DAB2, ELF1, G22P1, HNRPK, OAS1, RALB, TIMP2, XRCC5	Cancer; Cell Death; Reproductive System Disease

Pathway analysis of the 20 genes hypervariable under all growth conditions contained a network of 18 genes involved in TGFβ1 signaling. Contained in the 33 genes that were hypervariable on both ECM preparations but not on plastic were two networks of 15 and 11 genes respectively. The 15 gene network consisted of genes involved in TGFβ1 signaling, the AKT1 survival network, CDKN1A cell cycle regulation and IL1β signaling. The 11 gene network consisted of a c-Myc response network and EGF signaling. These genes are also listed in Table 4 and the pathways are presented [see Additional file 6].

### Transcriptional regulatory element (TRE) analysis of correlational clusters

**Figure 3:**
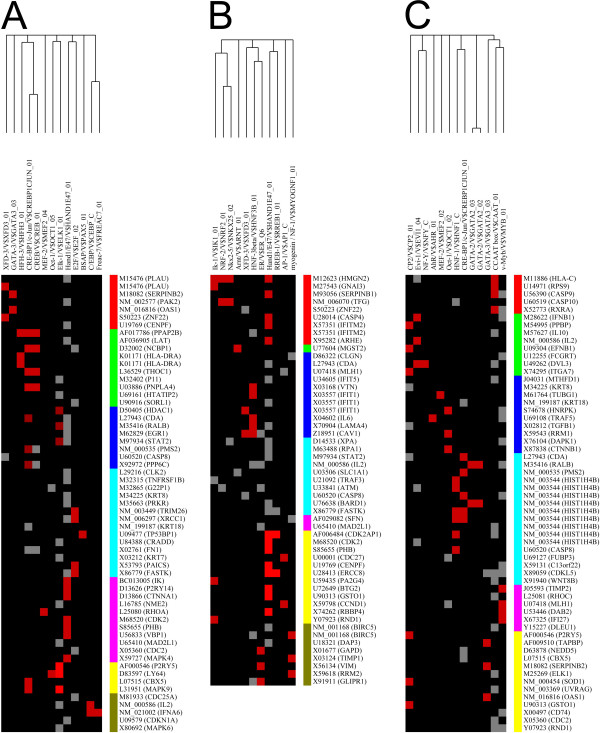
**Filtered maps of TREs on gene clusters of hypervariable genes identified in cells growing on different matrixes**. **A**, **B **and **C **show significantly significantly overrepresented (p < 0.05) TREs from cells grown on Matrigel, Plastic and SISgel, respectively. Colored bars represent individual gene clusters. The TREs and the transcription factors that bind to them (linked to Enterz Gene) are CCAAT/NFYC; CP2/TFCP2; CREB/CREBP; CRE-BP1/ATF2; E2F/E2F2; Elk-1/ELK1; ER/ESR1; GATA-3/GATA-3; Hand1/HAND1; HFH-3/FOXI1; HNF-1/TCF1; HNF-3/FOXA1; myogenin/MYOG; Oct-1/POU2F1; v-Myb/MYB; XFD-3/FOXA1.

TRE analysis was used to determine whether the correlation clustering could be interpreted in terms of the actions of networks of transcription factors. Figure 3 shows the results of analysis of TREs with the PAINT program according to the clusters defined by the hierarchical clustering. Each cluster expressed a pattern that was distinct from other clusters confirming the results of hierarchical clustering. This can be seen on full TREs maps [see Additional file 5] or, more clearly, on filtered maps, see Figure 3.

## Discussion

In this study we sought to identify genes that modulate the malignant phenotype of bladder cancer. The experimental design made genes modulating the phenotype hypervariable as six different cell lines with different inherent malignancies were grown on plastic and two different extracellular matrices. Because varying the substrate on which the cells are grown changes the malignant phenotype, genes that modulate the phenotype were therefore brought to the fore. Our approach for analysis of this noisy dataset first consisted of identifying the subset of hypervariable genes because this subset will contain the set of genes encapsulating the relevant biological effect [14]. This step is designed to minimize false negatives for further *in silico *analysis. The subsequent steps sought to generate testable hypotheses with minimal false positives. By finding gene ontologies, pathways and TRE motifs that were significantly enriched within individual clusters, genes that potentially functioned together in pathways were identified. Because microarray studies are weighted toward high expression genes, these approaches, particularly pathways and TRE analysis can identify effects due to low expression genes such as transcription factors whose expression may be too low to be detected with the microarray. Because these determinations are made with reference to statistical probability, the only step in the analysis process that does not contain a quantitative estimate of statistical significance is the clustering.

Except for clusters M4 and P7 the driving force for clustering appeared to represent the behavior of individual cell lines rather than overall malignancy. However, these two clusters identify genes generally expressed at higher levels in the malignant cells than in the HUC cells. Of particular interest is the apparent activation of pro-survival AKT1 signaling in the malignant cells on Matrigel [see Additional file 6]. Likewise, the receptor for TGFβ1 was expressed at high levels on all the malignant cells on Matrigel (Figure 2), which supports the finding of TGFβ1 signaling genes in several clusters by the pathway analysis tool. Other ontologies in these two clusters clearly showed connections to cancer and apoptosis. The gene ontology and pathway analysis agreed in identifying the p53 signaling and TGFβ1 pathways within these clusters. While the mechanisms identified as operating only within a single cell line are probably less interesting than those operating in several, identification of a range of behaviors in bladder cancers could become useful if pathway-specific drugs are developed.

Some 20 genes were identified as being hypervariable, regardless the substrate. These represent a group of genes that are not modulated by the ECM but are differentially expressed among the different cell lines. Examples include Prohibitin (PHB), a survival gene, which is entirely matrix-independent. The connection with AKT1 survival pathway is clear. In bladder cancer, activation of AKT1-mediated survival has only been shown in TCCSUP cells in culture [24]. AKT1 can cause resistance to therapy in other cancers [25]. Interestingly, genes coding for two polysaccharide-binding proteins were also present. That these two would be differentially expressed by different cell types growing on polysaccharide-containing gels and the minimal endogenous matrix secreted by cells growing on plastic is consistent with the role of these molecules in differentiation and growth [26]. Additionally, 33 genes were identified as being hypervariable on both Matrigel and SISgel, in spite of the suppression of many features of the malignant phenotype on the latter. These genes probably represent a "core" set of cancer genes. Ontologies for this cluster of genes were cancer, cell morphology and cell cycle networks. Pathway analysis showed fifteen of these genes functioned in TGFβ1 signaling, which is a potent regulator of extracellular matrix production and proliferation [27]. An important role for TGFβ1 in bladder cancer has been proposed [28]. Eleven of these 33 genes are interconnected within the MYC network. Deregulation of MYC genes is associated with several malignancies [29]. Although the ECM can have major effects on the biology of individual cancers, a core of oncogenes involved in the p53 and MYC networks is unaffected. Interestingly, a set of signature genes for the suppression of the malignant phenotype by SISgel was not observed. Either the correct probes are not on the array, or each cell type respond uniquely to a changing matrix. Previous results showed this response was not due to integrin signaling [9] as has been reported by Weaver, et. al [30] to govern phenotypic reversion of glandular breast cancers.

The TRE analysis confirmed the uniqueness of the clustering and suggests that a limited set of TREs may be driving gene expression in this study. Notable is Hand1, which appears in most of the genes. This gene has been identified as playing a role in heart development [31], but the abundance in these genes that are expressed in bladder suggests it may also act as a bladder-specific factor. Several genes overexpressed in malignant cell lines in cluster M4 carried the E2F TRE. The corresponding transcription factor has been shown to promote cell growth in common human carcinomas [32] and to be dependent on the p53 pathway [32,33] supporting its role in upregulating the cluster in cancer cells. A different set of TREs was identified in Cluster P7. An estrogen receptor (ER) and myogenin (MYOG) were separately associated within P7. This combination suggests loss of epithelial differentiation. Bladder cancer cells were earlier demonstrated to contain both functional androgen and estrogen receptors without regard to the sex of the donor [34,35] while myogenin was primary expressed in rhabdomyosarcomas [36,37]. Identification of myogenin expression in bladder cancer cell lines is novel, and further research will be needed to determine if this transcription factor is actually active.

Comparisons to other microarray studies are, in general, less informative because of wide differences among array technologies [38] and criteria to identify "significant" genes. Nonetheless the composition of Cluster M4, which is comprised of genes that are under-expressed in HUC cells in comparison to all the cancer cells, shows 3 of the 28 genes in the cluster corresponded to 3 of the 29 genes identified as being diagnostic for TCC in patient samples [39]. This association suggests this cluster might provide additional diagnostic genes which is strongly supported by the finding of the p53-responsive gene network. In addition, although the comparison was made difficult by a lack of accession numbers or universal gene symbols, considerable correspondence between the 32 genes identified as hypervariable in 3-dimensional growth was noted with the set of genes reported to differentially expressed between superficial and invasive TCC [40].

## Conclusion

The extracellular matrix on which cancer cells are grown has a major effect on gene expression. A core of 20 malignancy-related genes were not affected by matrix, and 33 were differentially expressed on 3-dimensional culture as opposed to plastic. Activation of the AKT1 survival pathway on Matrigel suggests this pathway could be relevant to clinical bladder cancer. The TGFβ1 signaling pathway plays an important role in the expression of the malignant phenotype. The p53 response network is confirmed to play a central role, as does c-MYC signaling. Evidence suggests the E2F TF drives some malignancy-related genes, with several other TFs being implicated as well. The identified pathways may provide new targets for detection and treatment therapies.

## List of abbreviations

ECM – extracellular matrix

GO – gene ontology

SIS – small intestine submucosa

TCC – transitional cell carcinoma

TF – transcription factor

TRE – transcription regulatory element

## Competing interests

The author(s) declare that they have no competing interests.

## Authors' contributions

MD performed overall analysis of hypervariable genes, prepared figures, tables and additional material and helped to draft and finalize the manuscript; KDK made the microarray studies, including isolation of mRNA and amplification and performed the preliminary data analysis; REH wrote the first draft of the manuscript and directed the overall study; RS assisted with the interpretation of the TRE analysis; NK performed preliminary clustering and identification of hypervariable genes; ID performed the data normalization and assisted with the interpretation of results; MBC directed the overall bioinformatics program and helped with the data interpretation. All authors read and approved the final manuscript.

## Pre-publication history

The pre-publication history for this paper can be accessed here:



## Supplementary Material

Additional File 1 Table 1. Description and ontology of each hypervariable gene grouped by clusters on different matrixes Click here for file

Additional File 2 Table 2. **Hypervariable genes commonly expressed between different matrixes and their ontologies**. GOTM results for this group of genes are shown at the bottom of the table. O:Observed gene number in the GO category; E:Expected gene number in the GO category; R:Ratio of enrichment for the GO category; P:Significance of enrichment for the GO category Click here for file

Additional File 3 Table 3. **Hypervariable genes commonly expressed between SISgel and Matrigel and their ontologies**. GOTM results for this group of genes are shown at the bottom of the table. O:Observed gene number in the GO category; E:Expected gene number in the GO category; R:Ratio of enrichment for the GO category; P:Significance of enrichment for the GO category Click here for file

Additional File 4 Table 4. **Genes driving significant ontologies on clusters on different matrixes**. O:Observed gene number in the GO category; E:Expected gene number in the GO category; R:Ratio of enrichment for the GO category; P:Significance of enrichment for the GO category Click here for file

Additional File 6 Figure 2. **Gene networks identified by Ingenuity analysis**. Pathways for Matrigel, cluster 4, Plastic, cluster 7, common genes between all three matrixes, common genes between Matrigel and SISgel are shown. Focus genes that map to the Global Molecular Network are displayed with bold text. User input genes are emphasized by gray color. For pathway legend see bottom of the figure. Click here for file

Additional File 5 Figure 1A, 1B, 1C. **Complete TREs maps for different matrixes**. A, B and C show all promoters for a given set of genes expressed on Matrigel, Plastic and SISgel, respectively. Colored bars represent individual gene clusters. Click here for file
