# Supplementary material for: Analysis of the interaction of extracellular matrix and phenotype of bladder cancer cells
Source: BMC Cancer. 2006 Jan 13;6:12. doi: 10.1186/1471-2407-6-12 (PMC1360102; doi:10.1186/1471-2407-6-12)
Supplement: Additional File 1 — Table 1. Description and ontology of each hypervariable gene grouped by clusters on different matrixes [file 1471-2407-6-12-S1.doc]

***Supplementary Table 1. Description of All Hypervariable Genes Clustered on Different Matrixes.***

Matrigel

Cluster M1

| ***GENBANK*** | ***SYMBOL*** | ***GENENAME*** | ***GENEONTOLOGY*** |
| --- | --- | --- | --- |
| NM_016816 | [OAS1](http://bioinfo.weizmann.ac.il/cards-bin/carddisp?OAS1) | 2',5'-oligoadenylate synthetase 1, 40/46kDa | ATP binding; RNA binding; cytoplasm; immune response; nucleobase, nucleoside, nucleotide and nucleic acid metabolism; nucleotidyltransferase activity; response to virus; transferase activity |
| NM_002577 | [PAK2](http://bioinfo.weizmann.ac.il/cards-bin/carddisp?PAK2) | p21 (CDKN1A)-activated kinase 2 | ATP binding; negative regulation of protein kinase activity; protein amino acid phosphorylation; protein serine/threonine kinase activity; signal transduction; transferase activity |
| U19769 | [CENPF](http://bioinfo.weizmann.ac.il/cards-bin/carddisp?CENPF) | centromere protein F, 350/400ka (mitosin) | DNA replication and chromosome cycle; kinetochore; nucleus; regulation of mitosis; spindle |
| U82169 | [FZD9](http://bioinfo.weizmann.ac.il/cards-bin/carddisp?FZD9) | frizzled homolog 9 (Drosophila) | G-protein coupled receptor activity; G-protein coupled receptor protein signaling pathway; Wnt receptor activity; development; frizzled signaling pathway; integral to membrane; neurogenesis; plasma membrane |
| X69550 | [ARHGDIA](http://bioinfo.weizmann.ac.il/cards-bin/carddisp?ARHGDIA) | Rho GDP dissociation inhibitor (GDI) alpha | GTPase activator activity; Rho GDP-dissociation inhibitor activity; Rho protein signal transduction; cytoplasm; negative regulation of cell adhesion; protein binding |
| X62744 | [HLA-DMA](http://bioinfo.weizmann.ac.il/cards-bin/carddisp?HLA-DMA) | major histocompatibility complex, class II, DM alpha | MHC class II receptor activity; antigen presentation, exogenous antigen; antigen processing, exogenous antigen via MHC class II; chaperone activity; detection of pest/pathogen/parasite; immune response; integral to membrane; protein binding |
| X65923 | [FAU](http://bioinfo.weizmann.ac.il/cards-bin/carddisp?FAU) | Finkel-Biskis-Reilly murine sarcoma virus (FBR-MuSV) ubiquitously expressed (fox derived); ribosomal protein S30 | RNA binding; protein biosynthesis; ribosome; structural constituent of ribosome |
| M18082 | [SERPINB2](http://bioinfo.weizmann.ac.il/cards-bin/carddisp?SERPINB2) | serine (or cysteine) proteinase inhibitor, clade B (ovalbumin), member 2 | anti-apoptosis; plasminogen activator activity; serine-type endopeptidase inhibitor activity |
| M15476 | [PLAU](http://bioinfo.weizmann.ac.il/cards-bin/carddisp?PLAU) | plasminogen activator, urokinase | blood coagulation; cell growth and/or maintenance; chemotaxis; chymotrypsin activity; extracellular space; hydrolase activity; negative regulation of blood coagulation; proteolysis and peptidolysis; signal transduction; trypsin activity; u-plasminogen activator activity |
| D13666 | [POSTN](http://bioinfo.weizmann.ac.il/cards-bin/carddisp?POSTN) | periostin, osteoblast specific factor | cell adhesion; extracellular matrix; heparin binding; protein binding; skeletal development |
| U22897 | [NDP52](http://bioinfo.weizmann.ac.il/cards-bin/carddisp?NDP52) | nuclear domain 10 protein | Nucleus; viral life cycle |
| S50223 | [ZNF22](http://bioinfo.weizmann.ac.il/cards-bin/carddisp?ZNF22) | zinc finger protein 22 (KOX 15) | DNA binding; nucleus; regulation of transcription; zinc ion binding; odontogenesis |
| NM_021086 | NTF6G | neurotrophin 6, gamma pseudogene |  |

Cluster M2

| ***GENBANK*** | ***SYMBOL*** | ***GENENAME*** | ***GENEONTOLOGY*** |
| --- | --- | --- | --- |
| NM_001118 | [ADCYAP1R1](http://bioinfo.weizmann.ac.il/cards-bin/carddisp?ADCYAP1R1) | adenylate cyclase activating polypeptide 1 (pituitary) receptor type I | G-protein coupled receptor activity; G-protein coupled receptor protein signaling pathway; integral to plasma membrane; receptor activity; spermatogenesis; vasoactive intestinal polypeptide receptor activity |
| K01171 | [HLA-DRA](http://bioinfo.weizmann.ac.il/cards-bin/carddisp?HLA-DRA) | major histocompatibility complex, class II, DR alpha | MHC class II receptor activity; antigen presentation, exogenous antigen; antigen processing, exogenous antigen via MHC class II; immune response; integral to plasma membrane |
| Y12394 | [KPNA3](http://bioinfo.weizmann.ac.il/cards-bin/carddisp?KPNA3) | karyopherin alpha 3 (importin alpha 4) | NLS-bearing substrate-nucleus import; intracellular protein transport; nuclear localization sequence binding; nuclear pore; nucleus; protein complex assembly; protein transporter activity |
| D32002 | [NCBP1](http://bioinfo.weizmann.ac.il/cards-bin/carddisp?NCBP1) | nuclear cap binding protein subunit 1, 80kDa | RNA cap binding; RNA splicing; mRNA processing; mRNA-nucleus export; nucleoplasm; protein biosynthesis; transport |
| U69161 | [HTATIP2](http://bioinfo.weizmann.ac.il/cards-bin/carddisp?HTATIP2) | HIV-1 Tat interactive protein 2, 30kDa | RNA polymerase II transcription factor activity; anti-apoptosis; cellular_component unknown; induction of apoptosis; molecular_function unknown; nucleus; protein binding; regulation of transcription from Pol II promoter; transcription coactivator activity |
| AF036905 | [LAT](http://bioinfo.weizmann.ac.il/cards-bin/carddisp?LAT) | linker for activation of T cells | SH3/SH2 adaptor protein activity; immune response; integral to membrane; signal transduction |
| U19713 | [AIF1](http://bioinfo.weizmann.ac.il/cards-bin/carddisp?AIF1) | allograft inflammatory factor 1 | biological_process unknown; calcium ion binding; cell cycle arrest; cellular_component unknown; inflammatory response; molecular_function unknown; negative regulation of cell proliferation; nucleus; response to stress |
| U03886 | [DXS1283E](http://bioinfo.weizmann.ac.il/cards-bin/carddisp?DXS1283E) | GS2 gene | biological_process unknown; catalytic activity; cellular_component unknown; molecular_function unknown; nutrient reservoir activity |
| AF017786 | [PPAP2B](http://bioinfo.weizmann.ac.il/cards-bin/carddisp?PPAP2B) | phosphatidic acid phosphatase type 2B | cell growth and/or maintenance; germ-cell migration; hydrolase activity; lipid metabolism; membrane; phosphoprotein phosphatase activity |
| U53446 | [DAB2](http://bioinfo.weizmann.ac.il/cards-bin/carddisp?DAB2) | disabled homolog 2, mitogen-responsive phosphoprotein (Drosophila) | cell proliferation |
| U90916 | [SORL1](http://bioinfo.weizmann.ac.il/cards-bin/carddisp?SORL1) | sortilin-related receptor, L(DLR class) A repeats-containing | cholesterol metabolism; integral to plasma membrane; internalization receptor activity; lipid transport; lipid transporter activity; receptor mediated endocytosis; transmembrane receptor activity |
| D16234 | [GRP58](http://bioinfo.weizmann.ac.il/cards-bin/carddisp?GRP58) | glucose regulated protein, 58kDa | cysteine-type endopeptidase activity; electron transport; electron transporter activity; endoplasmic reticulum; isomerase activity; phospholipase C activity; protein disulfide isomerase activity; protein-ER retention; protein-nucleus import; signal transduction |
| U07231 | [GRSF1](http://bioinfo.weizmann.ac.il/cards-bin/carddisp?GRSF1) | G-rich RNA sequence binding factor 1 | cytoplasm; mRNA binding; mRNA polyadenylation; nucleic acid binding |
| M32402 | [P11](http://bioinfo.weizmann.ac.il/cards-bin/carddisp?P11) | 26 serine protease | extracellular space; hydrolase activity; proteolysis and peptidolysis; serine-type endopeptidase activity |
| D26067 | [KIAA0033](http://bioinfo.weizmann.ac.il/cards-bin/carddisp?KIAA0033) | KIAA0033 protein | sugar porter activity; phosphoenolpyruvate-dependent sugar phosphotransferase system |
| L36529 | [THOC1](http://bioinfo.weizmann.ac.il/cards-bin/carddisp?THOC1) | THO complex 1 | nuclear mRNA splicing; DNA binding; RNA binding; structural molecule activity; protein binding |
| X63679 | [TRAM1](http://bioinfo.weizmann.ac.il/cards-bin/carddisp?TRAM1) | translocation associated membrane protein 1 | receptor activity; endoplasmic reticulum; protein targeting; cotranslational protein-membrane targeting; protein transport |

Cluster M3

| ***GENBANK*** | ***SYMBOL*** | ***GENENAME*** | ***GENEONTOLOGY*** |
| --- | --- | --- | --- |
| NM_000535 | [PMS2](http://bioinfo.weizmann.ac.il/cards-bin/carddisp?PMS2) | PMS2 postmeiotic segregation increased 2 (S. cerevisiae) | ATP binding; DNA binding; mismatch repair; negative regulation of cell cycle; nucleus |
| NM_000534 | [PMS1](http://bioinfo.weizmann.ac.il/cards-bin/carddisp?PMS1) | PMS1 postmeiotic segregation increased 1 (S. cerevisiae) | ATP binding; DNA binding; mismatch repair; negative regulation of cell cycle; nucleus; regulation of transcription, DNA-dependent |
| NM_005347 | [HSPA5](http://bioinfo.weizmann.ac.il/cards-bin/carddisp?HSPA5) | heat shock 70kDa protein 5 (glucose-regulated protein, 78kDa) | ATP binding; Hsp70/Hsp90 organizing protein activity; endoplasmic reticulum lumen |
| U69127 | [FUBP3](http://bioinfo.weizmann.ac.il/cards-bin/carddisp?FUBP3) | far upstream element (FUSE) binding protein 3 | DNA binding; nucleus; regulation of transcription, DNA-dependent |
| M96684 | [PURA](http://bioinfo.weizmann.ac.il/cards-bin/carddisp?PURA) | purine-rich element binding protein A | DNA replication initiation; RNA polymerase II transcription factor activity, enhancer binding; nucleus; regulation of transcription, DNA-dependent; single-stranded DNA binding |
| X92972 | [PPP6C](http://bioinfo.weizmann.ac.il/cards-bin/carddisp?PPP6C) | protein phosphatase 6, catalytic subunit | G1/S transition of mitotic cell cycle; hydrolase activity; manganese ion binding; protein amino acid dephosphorylation; protein serine/threonine phosphatase activity |
| M35416 | [RALB](http://bioinfo.weizmann.ac.il/cards-bin/carddisp?RALB) | v-ral simian leukemia viral oncogene homolog B (ras related; GTP binding protein) | GTP binding; signal transduction; small GTPase mediated signal transduction; small monomeric GTPase activity |
| M97934 | [STAT2](http://bioinfo.weizmann.ac.il/cards-bin/carddisp?STAT2) | signal transducer and activator of transcription 2, 113kDa | JAK-STAT cascade; hematopoietin/interferon-class (D200-domain) cytokine receptor signal transducer activity; intracellular signaling cascade; nucleus; regulation of transcription from Pol II promoter; signal transducer activity; transcription factor activity |
| D50405 | [HDAC1](http://bioinfo.weizmann.ac.il/cards-bin/carddisp?HDAC1) | histone deacetylase 1 | anti-apoptosis; chromatin modification; cytoplasm; histone deacetylase activity; histone deacetylase complex; histone deacetylation; hydrolase activity; nucleus; regulation of transcription, DNA-dependent; transcription factor activity; transcription factor binding |
| U60520 | [CASP8](http://bioinfo.weizmann.ac.il/cards-bin/carddisp?CASP8) | caspase 8, apoptosis-related cysteine protease | apoptotic program; caspase activity; cysteine-type peptidase activity; cytoskeleton; hydrolase activity; mitochondrion; protein binding; proteolysis and peptidolysis; regulation of apoptosis |
| X91940 | [WNT8B](http://bioinfo.weizmann.ac.il/cards-bin/carddisp?WNT8B) | wingless-type MMTV integration site family, member 8B | cell-cell signaling; development; extracellular; frizzled-2 signaling pathway; neurogenesis; signal transducer activity; signal transduction |
| L27943 | [CDA](http://bioinfo.weizmann.ac.il/cards-bin/carddisp?CDA) | cytidine deaminase | cytidine deaminase activity; cytidine metabolism; hydrolase activity; nucleobase, nucleoside, nucleotide and nucleic acid metabolism; zinc ion binding |
| M62829 | [EGR1](http://bioinfo.weizmann.ac.il/cards-bin/carddisp?EGR1) | early growth response 1 | nucleus; regulation of transcription, DNA-dependent; transcription factor activity |
| M82882 | [ELF1](http://bioinfo.weizmann.ac.il/cards-bin/carddisp?ELF1) | E74-like factor 1 (ets domain transcription factor) | nucleus; regulation of transcription, DNA-dependent; transcription factor activity; transcriptional activator activity |

Cluster M4

| ***GENBANK*** | ***SYMBOL*** | ***GENENAME*** | ***GENEONTOLOGY*** |
| --- | --- | --- | --- |
| M63167 | [AKT1](http://bioinfo.weizmann.ac.il/cards-bin/carddisp?AKT1) | v-akt murine thymoma viral oncogene homolog 1 | ATP binding; G-protein coupled receptor protein signaling pathway; anti-apoptosis; nitric oxide biosynthesis; nucleus; protein amino acid phosphorylation; receptor signaling protein serine/threonine kinase activity; response to heat; signal transduction; transferase activity |
| X76104 | [DAPK1](http://bioinfo.weizmann.ac.il/cards-bin/carddisp?DAPK1) | death-associated protein kinase 1 | ATP binding; actin cytoskeleton; apoptosis; calcium/calmodulin-dependent protein kinase activity; calmodulin binding; calmodulin-dependent protein kinase I activity; induction of apoptosis by extracellular signals; protein amino acid phosphorylation; protein kinase cascade; protein serine/threonine kinase activity; signal transduction; transferase activity |
| M35663 | [PRKR](http://bioinfo.weizmann.ac.il/cards-bin/carddisp?PRKR) | protein kinase, interferon-inducible double stranded RNA dependent | ATP binding; apoptosis; cell cycle; double-stranded RNA binding; eukaryotic translation initiation factor 2alpha kinase activity; immune response; intracellular; negative regulation of cell proliferation; protein amino acid phosphorylation; protein phosphatase type 2A regulator activity; protein serine/threonine kinase activity; regulation of transcription, DNA-dependent; transferase activity |
| L29216 | [CLK2](http://bioinfo.weizmann.ac.il/cards-bin/carddisp?CLK2) | CDC-like kinase 2 | ATP binding; nucleus; protein amino acid phosphorylation; protein serine/threonine kinase activity; protein-tyrosine kinase activity; transferase activity |
| M32865 | [G22P1](http://bioinfo.weizmann.ac.il/cards-bin/carddisp?G22P1) | thyroid autoantigen 70kDa (Ku antigen) | ATP-dependent DNA helicase activity; DNA ligation; double-strand break repair via nonhomologous end-joining; double-stranded DNA binding; helicase activity; membrane fraction; nucleus |
| S74678 | [HNRPK](http://bioinfo.weizmann.ac.il/cards-bin/carddisp?HNRPK) | heterogeneous nuclear ribonucleoprotein K | DNA binding; RNA binding; RNA processing; heterogeneous nuclear ribonucleoprotein complex; nucleoplasm |
| NM_003449 | [TRIM26](http://bioinfo.weizmann.ac.il/cards-bin/carddisp?TRIM26) | tripartite motif-containing 26 | DNA binding; intracellular; protein binding; zinc ion binding |
| M29870 | [RAC1](http://bioinfo.weizmann.ac.il/cards-bin/carddisp?RAC1) | ras-related C3 botulinum toxin substrate 1 (rho family, small GTP binding protein Rac1) | GTP binding; cell adhesion; cell motility; inflammatory response; morphogenesis; small GTPase mediated signal transduction; small monomeric GTPase activity |
| U09477 | [TP53BP1](http://bioinfo.weizmann.ac.il/cards-bin/carddisp?TP53BP1) | tumor protein p53 binding protein, 1 | P-element binding; cytoplasm; nucleoplasm; positive regulation of transcription, DNA-dependent; protein binding; signal transduction; transcriptional activator activity |
| X02761 | [FN1](http://bioinfo.weizmann.ac.il/cards-bin/carddisp?FN1) | fibronectin 1 | acute-phase response; cell adhesion; cell migration; collagen binding; extracellular; extracellular matrix structural constituent; heparin binding; response to wounding |
| X02812 | [TGFB1](http://bioinfo.weizmann.ac.il/cards-bin/carddisp?TGFB1) | transforming growth factor, beta 1 (Camurati-Engelmann disease) | anti-apoptosis; cell growth; cell proliferation; cell-cell signaling; growth; regulation of cell cycle; transforming growth factor beta receptor binding; transforming growth factor beta receptor signaling pathway |
| U33286 | [CSE1L](http://bioinfo.weizmann.ac.il/cards-bin/carddisp?CSE1L) | CSE1 chromosome segregation 1-like (yeast) | apoptosis; cell proliferation; cytoplasm; importin-alpha export receptor activity; intracellular protein transport; nuclear pore; nucleus; protein transporter activity; protein-nucleus import, docking |
| X86779 | [FASTK](http://bioinfo.weizmann.ac.il/cards-bin/carddisp?FASTK) | FAST kinase | apoptosis; induction of apoptosis by extracellular signals; kinase activity; protein amino acid phosphorylation; protein serine/threonine kinase activity; signal transduction; transferase activity |
| M32315 | [TNFRSF1B](http://bioinfo.weizmann.ac.il/cards-bin/carddisp?TNFRSF1B) | tumor necrosis factor receptor superfamily, member 1B | apoptosis; integral to membrane; receptor activity; tumor necrosis factor receptor activity |
| U60519 | [CASP10](http://bioinfo.weizmann.ac.il/cards-bin/carddisp?CASP10) | caspase 10, apoptosis-related cysteine protease | caspase activity; cysteine-type peptidase activity; hydrolase activity; induction of apoptosis; peptidase activity; protein binding; proteolysis and peptidolysis; regulation of apoptosis |
| M30938 | [XRCC5](http://bioinfo.weizmann.ac.il/cards-bin/carddisp?XRCC5) | X-ray repair complementing defective repair in Chinese hamster cells 5 (double-strand-break rejoining; Ku autoantigen, 80kDa) | cellular_component unknown; molecular_function unknown; regulation of DNA repair |
| M34225 | [KRT8](http://bioinfo.weizmann.ac.il/cards-bin/carddisp?KRT8) | keratin 8 | cytoskeleton organization and biogenesis; intermediate filament; phosphorylation; structural molecule activity |
| X03212 | [KRT7](http://bioinfo.weizmann.ac.il/cards-bin/carddisp?KRT7) | keratin 7 | cytoskeleton organization and biogenesis; intermediate filament; structural molecule activity |
| NM_006297 | [XRCC1](http://bioinfo.weizmann.ac.il/cards-bin/carddisp?XRCC1) | X-ray repair complementing defective repair in Chinese hamster cells 1 | damaged DNA binding; nucleus; single strand break repair |
| U65928 | [COPS5](http://bioinfo.weizmann.ac.il/cards-bin/carddisp?COPS5) | COP9 constitutive photomorphogenic homolog subunit 5 (Arabidopsis) | eukaryotic translation initiation factor 3 complex; protein biosynthesis; transcription coactivator activity; transcription from Pol II promoter; translation initiation factor activity |
| J05593 | [TIMP2](http://bioinfo.weizmann.ac.il/cards-bin/carddisp?TIMP2) | tissue inhibitor of metalloproteinase 2 | extracellular matrix; metalloendopeptidase inhibitor activity |
| U84388 | [CRADD](http://bioinfo.weizmann.ac.il/cards-bin/carddisp?CRADD) | CASP2 and RIPK1 domain containing adaptor with death domain | induction of apoptosis via death domain receptors; intracellular; protein binding; regulation of apoptosis; signal transduction |
| M18112 | [ADPRT](http://bioinfo.weizmann.ac.il/cards-bin/carddisp?ADPRT) | ADP-ribosyltransferase (NAD+; poly (ADP-ribose) polymerase) | DNA binding; NAD+ ADP-ribosyltransferase activity; nucleus; DNA repair; transcription from RNA polymerase II promoter |
| NM_004323 | [BAG1](http://bioinfo.weizmann.ac.il/cards-bin/carddisp?BAG1) | BCL2-associated athanogene | receptor signaling protein activity; cytoplasm; protein folding; protein modification; apoptosis |
| U78798 | [TRAF6](http://bioinfo.weizmann.ac.il/cards-bin/carddisp?TRAF6) | TNF receptor-associated factor 6 | ubiquitin ligase complex; ubiquitin-protein ligase activity; signal transducer activity; protein binding; signal transduction |
| Y08614 | [XPO1](http://bioinfo.weizmann.ac.il/cards-bin/carddisp?XPO1) | exportin 1 (CRM1 homolog, yeast) | protein-nucleus import; binding; nuclear pore; nucleoplasm; cytoplasm |
| NM_199187 | [KRT18](http://bioinfo.weizmann.ac.il/cards-bin/carddisp?KRT18) | keratin 18 | structural constituent of cytoskeleton; intermediate filament; morphogenesis |
| X53793 | [PAICS](http://bioinfo.weizmann.ac.il/cards-bin/carddisp?PAICS) | phosphoribosylaminoimidazole carboxylase, phosphoribosylaminoimidazole succinocarboxamide synthetase | phosphoribosylaminoimidazole carboxylase activity; phosphoribosylaminoimidazolesuccinocarboxamide synthase activity; purine nucleotide biosynthesis; 'de novo' IMP biosynthesis; purine base biosynthesis |
| AF022385 | [PDCD10](http://bioinfo.weizmann.ac.il/cards-bin/carddisp?PDCD10) | programmed cell death 10 |  |

Cluster M5

| ***GENBANK*** | ***SYMBOL*** | ***GENENAME*** | ***GENEONTOLOGY*** |
| --- | --- | --- | --- |
| M33336 | [PRKAR1A](http://bioinfo.weizmann.ac.il/cards-bin/carddisp?PRKAR1A) | protein kinase, cAMP-dependent, regulatory, type I, alpha (tissue specific extinguisher 1) | 3',5'-cAMP binding; cAMP-dependent protein kinase complex; cAMP-dependent protein kinase regulator activity; intracellular signaling cascade; protein amino acid phosphorylation; regulation of transcription from Pol II promoter |
| L16785 | [NME2](http://bioinfo.weizmann.ac.il/cards-bin/carddisp?NME2) | non-metastatic cells 2, protein (NM23B) expressed in | ATP binding; CTP biosynthesis; GTP biosynthesis; UTP biosynthesis; kinase activity; negative regulation of cell cycle; negative regulation of cell proliferation; nucleoside triphosphate biosynthesis; nucleoside-diphosphate kinase activity; nucleus; regulation of transcription, DNA-dependent; transcription factor activity; transferase activity |
| M68520 | [CDK2](http://bioinfo.weizmann.ac.il/cards-bin/carddisp?CDK2) | cyclin-dependent kinase 2 | ATP binding; G2/M transition of mitotic cell cycle; cell cycle; cyclin-dependent protein kinase activity; cytokinesis; cytoplasm; mitosis; nucleus; positive regulation of cell proliferation; protein amino acid phosphorylation; protein serine/threonine kinase activity; regulation of DNA replication; transferase activity; traversing start control point of mitotic cell cycle |
| X59727 | [MAPK4](http://bioinfo.weizmann.ac.il/cards-bin/carddisp?MAPK4) | mitogen-activated protein kinase 4 | ATP binding; MAP kinase activity; cell cycle; protein amino acid phosphorylation; protein serine/threonine kinase activity; transferase activity |
| X05360 | [CDC2](http://bioinfo.weizmann.ac.il/cards-bin/carddisp?CDC2) | cell division cycle 2, G1 to S and G2 to M | ATP binding; cyclin-dependent protein kinase activity; cytokinesis; mitosis; nucleus; protein amino acid phosphorylation; transferase activity; traversing start control point of mitotic cell cycle |
| S85655 | [PHB](http://bioinfo.weizmann.ac.il/cards-bin/carddisp?PHB) | prohibitin | DNA metabolism; cell growth and/or maintenance |
| L22005 | [CDC34](http://bioinfo.weizmann.ac.il/cards-bin/carddisp?CDC34) | cell division cycle 34 | DNA replication initiation; G1/S transition of mitotic cell cycle; ligase activity; nucleus; ubiquitin conjugating enzyme activity; ubiquitin cycle; ubiquitin-protein ligase activity |
| D13626 | [GPR105](http://bioinfo.weizmann.ac.il/cards-bin/carddisp?GPR105) | G protein-coupled receptor 105 | G-protein coupled receptor protein signaling pathway; UDP-activated nucleotide receptor activity; integral to membrane; rhodopsin-like receptor activity |
| U22398 | [CDKN1C](http://bioinfo.weizmann.ac.il/cards-bin/carddisp?CDKN1C) | cyclin-dependent kinase inhibitor 1C (p57, Kip2) | G1 phase of mitotic cell cycle; cell cycle; cell cycle arrest; cyclin-dependent protein kinase inhibitor activity; negative regulation of cell cycle; negative regulation of cell proliferation; nucleus; regulation of CDK activity |
| L25080 | [ARHA](http://bioinfo.weizmann.ac.il/cards-bin/carddisp?ARHA) | ras homolog gene family, member A | GTP binding; Rho protein signal transduction; Rho small monomeric GTPase activity; actin cytoskeleton organization and biogenesis; cell growth and/or maintenance; cytoskeleton; membrane |
| D13866 | [CTNNA1](http://bioinfo.weizmann.ac.il/cards-bin/carddisp?CTNNA1) | catenin (cadherin-associated protein), alpha 1, 102kDa | cell adhesion; cytoskeleton; protein binding; structural molecule activity |
| M55172 | [AGC1](http://bioinfo.weizmann.ac.il/cards-bin/carddisp?AGC1) | aggrecan 1 (chondroitin sulfate proteoglycan 1, large aggregating proteoglycan, antigen identified by monoclonal antibody A0122) | cell adhesion; extracellular matrix; heterophilic cell adhesion; hyaluronic acid binding; sugar binding |
| U66469 | [CGRRF1](http://bioinfo.weizmann.ac.il/cards-bin/carddisp?CGRRF1) | cell growth regulator with ring finger domain 1 | cell cycle arrest; negative regulation of cell proliferation; response to stress |
| U47413 | [CCNG1](http://bioinfo.weizmann.ac.il/cards-bin/carddisp?CCNG1) | cyclin G1 | cell cycle; cytokinesis; mitosis; nucleus; regulation of CDK activity |
| U65410 | [MAD2L1](http://bioinfo.weizmann.ac.il/cards-bin/carddisp?MAD2L1) | MAD2 mitotic arrest deficient-like 1 (yeast) | cell cycle; kinetochore; mitosis; mitotic checkpoint; nucleus |
| X02811 | [PDGFB](http://bioinfo.weizmann.ac.il/cards-bin/carddisp?PDGFB) | platelet-derived growth factor beta polypeptide (simian sarcoma viral (v-sis) oncogene homolog) | cell proliferation; extracellular; growth factor activity; membrane; platelet-derived growth factor receptor binding; regulation of cell cycle; response to wounding |
| BC013005 | [IK](http://bioinfo.weizmann.ac.il/cards-bin/carddisp?IK) | IK cytokine, down-regulator of HLA II | cell-cell signaling; cytokine activity; extracellular space; immune response; soluble fraction |
| BC031020 | [IFI30](http://bioinfo.weizmann.ac.il/cards-bin/carddisp?IFI30) | interferon, gamma-inducible protein 30 | extracellular; immune response; lysosome; oxidoreductase activity |
| M96956 | [TDGF3](http://bioinfo.weizmann.ac.il/cards-bin/carddisp?TDGF3) | teratocarcinoma-derived growth factor 3, pseudogene | mesoderm cell fate determination; growth factor activity; cellular_component unknown |
| U56833 | [VBP1](http://bioinfo.weizmann.ac.il/cards-bin/carddisp?VBP1) | von Hippel-Lindau binding protein 1 | protein folding; prefoldin complex; unfolded protein binding |

Cluster M6

| ***GENBANK*** | ***SYMBOL*** | ***GENENAME*** | ***GENEONTOLOGY*** |
| --- | --- | --- | --- |
| D83597 | [LY64](http://bioinfo.weizmann.ac.il/cards-bin/carddisp?LY64) | lymphocyte antigen 64 homolog, radioprotective 105kDa (mouse) | immune response; inflammatory response; integral to membrane; plasma membrane; protein binding; receptor activity |
| L07515 | [CBX5](http://bioinfo.weizmann.ac.il/cards-bin/carddisp?CBX5) | chromobox homolog 5 (HP1 alpha homolog, Drosophila) | chromatin; chromatin assembly/disassembly; chromatin binding; nuclear heterochromatin; nuclear membrane |
| AF000546 | [P2RY5](http://bioinfo.weizmann.ac.il/cards-bin/carddisp?P2RY5) | purinergic receptor P2Y, G-protein coupled, 5 | G-protein coupled receptor protein signaling pathway; integral to membrane; purinergic nucleotide receptor activity, G-protein coupled; rhodopsin-like receptor activity |
| L31951 | [MAPK9](http://bioinfo.weizmann.ac.il/cards-bin/carddisp?MAPK9) | mitogen-activated protein kinase 9 | ATP binding; JNK cascade; JUN kinase activity; MAP kinase activity; nucleus; protein amino acid phosphorylation; protein serine/threonine kinase activity; response to stress; signal transduction; transferase activity |
| U78095 | [SPINT2](http://bioinfo.weizmann.ac.il/cards-bin/carddisp?SPINT2) | serine protease inhibitor, Kunitz type, 2 | cell motility; extracellular; integral to membrane; serine-type endopeptidase inhibitor activity; soluble fraction |

Cluster M7

| ***GENBANK*** | ***SYMBOL*** | ***GENENAME*** | ***GENEONTOLOGY*** |
| --- | --- | --- | --- |
| X80692 | [MAPK6](http://bioinfo.weizmann.ac.il/cards-bin/carddisp?MAPK6) | mitogen-activated protein kinase 6 | ATP binding; MAP kinase activity; cell cycle; protein amino acid phosphorylation; protein serine/threonine kinase activity; signal transduction; transferase activity |
| L29220 | [CLK3](http://bioinfo.weizmann.ac.il/cards-bin/carddisp?CLK3) | CDC-like kinase 3 | ATP binding; nucleus; protein amino acid phosphorylation; protein serine/threonine kinase activity; protein-tyrosine kinase activity; transferase activity |
| M57627 | [IL10](http://bioinfo.weizmann.ac.il/cards-bin/carddisp?IL10) | interleukin 10 | B-cell differentiation; B-cell proliferation; T-helper 2 type immune response; anti-apoptosis; cell-cell signaling; cytokine activity; cytoplasmic sequestering of NF-kappaB; extracellular; hemopoiesis; immune cell chemotaxis; immune response; interleukin-10 receptor binding; negative regulation of MHC class II biosynthesis; negative regulation of T-cell proliferation; negative regulation of interferon-alpha biosynthesis; negative regulation of interferon-gamma biosynthesis; negative regulation of nitric oxide biosynthesis; regulation of isotype switching |
| M13194 | [ERCC1](http://bioinfo.weizmann.ac.il/cards-bin/carddisp?ERCC1) | excision repair cross-complementing rodent repair deficiency, complementation group 1 (includes overlapping antisense sequence) | DNA repair; damaged DNA binding; endodeoxyribonuclease activity; endonuclease activity; hydrolase activity; morphogenesis; nucleotide-excision repair; nucleus |
| L25081 | [ARHC](http://bioinfo.weizmann.ac.il/cards-bin/carddisp?ARHC) | ras homolog gene family, member C | GTP binding; Rho small monomeric GTPase activity; catalytic activity; cell growth and/or maintenance; small GTPase mediated signal transduction |
| M81934 | [CDC25B](http://bioinfo.weizmann.ac.il/cards-bin/carddisp?CDC25B) | cell division cycle 25B | M phase of mitotic cell cycle; cytokinesis; hydrolase activity; intracellular; mitosis; positive regulation of cell proliferation; protein amino acid dephosphorylation; protein-tyrosine-phosphatase activity; regulation of cell cycle |
| M11886 | [HLA-C](http://bioinfo.weizmann.ac.il/cards-bin/carddisp?HLA-C) | major histocompatibility complex, class I, C | MHC class I receptor activity; MHC class II receptor activity; antigen presentation, endogenous antigen; antigen processing, endogenous antigen via MHC class I; immune response; integral to membrane |
| X74295 | [MGC17301](http://bioinfo.weizmann.ac.il/cards-bin/carddisp?MGC17301) | hypothetical protein MGC17301 | S-adenosylmethionine-dependent methyltransferase activity |
| NM_000586 | [IL2](http://bioinfo.weizmann.ac.il/cards-bin/carddisp?IL2) | interleukin 2 | T-cell differentiation; anti-apoptosis; antimicrobial humoral response (sensu Vertebrata); cell adhesion; cell growth and/or maintenance; cell-cell signaling; cytokine activity; extracellular space; immune response; interleukin-2 receptor binding; kinase activator activity; natural killer cell activation; positive regulation of cell growth; positive regulation of cell proliferation |
| X02851 | [IL1A](http://bioinfo.weizmann.ac.il/cards-bin/carddisp?IL1A) | interleukin 1, alpha | anti-apoptosis; apoptosis; cell proliferation; cell-cell signaling; chemotaxis; cytoplasm; extracellular space; immune response; inflammatory response; interleukin-1 receptor binding; negative regulation of cell proliferation; regulation of cell cycle; signal transducer activity |
| K02770 | [IL1B](http://bioinfo.weizmann.ac.il/cards-bin/carddisp?IL1B) | interleukin 1, beta | antimicrobial humoral response (sensu Vertebrata); apoptosis; cell proliferation; cell-cell signaling; extracellular space; immune response; inflammatory response; interleukin-1 receptor binding; negative regulation of cell proliferation; regulation of cell cycle; signal transducer activity; signal transduction |
| U56390 | [CASP9](http://bioinfo.weizmann.ac.il/cards-bin/carddisp?CASP9) | caspase 9, apoptosis-related cysteine protease | apoptotic program; caspase activation via cytochrome c; caspase activity; enzyme activator activity; intracellular; protein binding; proteolysis and peptidolysis; regulation of apoptosis |
| X57766 | [MMP11](http://bioinfo.weizmann.ac.il/cards-bin/carddisp?MMP11) | matrix metalloproteinase 11 (stromelysin 3) | calcium ion binding; collagen catabolism; extracellular matrix; hydrolase activity; morphogenesis; stromelysin 3 activity; zinc ion binding |
| X03168 | [VTN](http://bioinfo.weizmann.ac.il/cards-bin/carddisp?VTN) | vitronectin (serum spreading factor, somatomedin B, complement S-protein) | cell adhesion; extracellular space; heparin binding; immune response; protein binding |
| U09579 | [CDKN1A](http://bioinfo.weizmann.ac.il/cards-bin/carddisp?CDKN1A) | cyclin-dependent kinase inhibitor 1A (p21, Cip1) | cell cycle arrest; cyclin-dependent protein kinase inhibitor activity; induction of apoptosis by intracellular signals; kinase activity; negative regulation of cell proliferation; nucleus; protein kinase activity; regulation of CDK activity |
| X06374 | [PDGFA](http://bioinfo.weizmann.ac.il/cards-bin/carddisp?PDGFA) | platelet-derived growth factor alpha polypeptide | cell proliferation; cell surface receptor linked signal transduction; cell-cell signaling; extracellular space; growth factor activity; membrane; platelet-derived growth factor receptor binding; regulation of cell cycle |
| X74295 | [ITGA7](http://bioinfo.weizmann.ac.il/cards-bin/carddisp?ITGA7) | integrin, alpha 7 | cell-matrix adhesion; cellular morphogenesis; homophilic cell adhesion; integrin complex; integrin-mediated signaling pathway; muscle development; protein binding; receptor activity |
| M81933 | [CDC25A](http://bioinfo.weizmann.ac.il/cards-bin/carddisp?CDC25A) | cell division cycle 25A | cellular_component unknown; cytokinesis; hydrolase activity; intracellular; mitosis; protein amino acid dephosphorylation; protein-tyrosine-phosphatase activity; regulation of CDK activity |
| NM_021002 | [IFNA6](http://bioinfo.weizmann.ac.il/cards-bin/carddisp?IFNA6) | interferon, alpha 6 | cellular_component unknown; defense response; extracellular; hematopoietin/interferon-class (D200-domain) cytokine receptor binding; response to virus |
| X03124 | [TIMP1](http://bioinfo.weizmann.ac.il/cards-bin/carddisp?TIMP1) | tissue inhibitor of metalloproteinase 1 (erythroid potentiating activity, collagenase inhibitor) | development; extracellular matrix; metalloendopeptidase inhibitor activity; metallopeptidase activity; positive regulation of cell proliferation; proteolysis and peptidolysis |

Plastic

Cluster P1

| ***GENBANK*** | ***SYMBOL*** | ***GENENAME*** | ***GENEONTOLOGY*** |
| --- | --- | --- | --- |
| M12623 | [HMGN2](http://bioinfo.weizmann.ac.il/cards-bin/carddisp?HMGN2) | high-mobility group nucleosomal binding domain 2 | DNA binding; chromatin; establishment and/or maintenance of chromatin architecture; nucleus; regulation of transcription, DNA-dependent |
| X95282 | [ARHE](http://bioinfo.weizmann.ac.il/cards-bin/carddisp?ARHE) | ras homolog gene family, member E | GTP binding; Rho small monomeric GTPase activity; actin cytoskeleton organization and biogenesis; cell adhesion; small GTPase mediated signal transduction |
| NM_006070 | [TFG](http://bioinfo.weizmann.ac.il/cards-bin/carddisp?TFG) | TRK-fused gene | biological_process unknown; cell growth and/or maintenance; cellular_component unknown; molecular_function unknown |
| U28014 | [CASP4](http://bioinfo.weizmann.ac.il/cards-bin/carddisp?CASP4) | caspase 4, apoptosis-related cysteine protease | caspase activity; cysteine-type peptidase activity; cytoplasm; hydrolase activity; induction of apoptosis; intracellular; protein binding; proteolysis and peptidolysis; regulation of apoptosis |
| NM_016335 | [PRODH](http://bioinfo.weizmann.ac.il/cards-bin/carddisp?PRODH) | proline dehydrogenase (oxidase) 1 | cellular_component unknown; glutamate biosynthesis; induction of apoptosis by oxidative stress; molecular_function unknown; proline catabolism; proline dehydrogenase activity |
| M93056 | [SERPINB1](http://bioinfo.weizmann.ac.il/cards-bin/carddisp?SERPINB1) | serine (or cysteine) proteinase inhibitor, clade B (ovalbumin), member 1 | cytoplasm; serine-type endopeptidase inhibitor activity |
| X57351 | [IFITM2](http://bioinfo.weizmann.ac.il/cards-bin/carddisp?IFITM2) | interferon induced transmembrane protein 2 (1-8D) | immune response; integral to membrane |
| M27543 | [GNAI3](http://bioinfo.weizmann.ac.il/cards-bin/carddisp?GNAI3) | guanine nucleotide binding protein (G protein), alpha inhibiting activity polypeptide 3 | GTPase activity; signal transducer activity; GTP binding; transport; signal transduction |
| S50223 | [ZNF22](http://bioinfo.weizmann.ac.il/cards-bin/carddisp?ZNF22) | zinc finger protein 22 (KOX 15) | DNA binding; nucleus; regulation of transcription; zinc ion binding; odontogenesis |

Cluster P2

| ***GENBANK*** | ***SYMBOL*** | ***GENENAME*** | ***GENEONTOLOGY*** |
| --- | --- | --- | --- |
| L20688 | [ARHGDIB](http://bioinfo.weizmann.ac.il/cards-bin/carddisp?ARHGDIB) | Rho GDP dissociation inhibitor (GDI) beta | GTPase activator activity; Rho GDP-dissociation inhibitor activity; Rho protein signal transduction; actin cytoskeleton organization and biogenesis; cytoplasmic vesicle; development; immune response; negative regulation of cell adhesion |
| U77604 | [MGST2](http://bioinfo.weizmann.ac.il/cards-bin/carddisp?MGST2) | microsomal glutathione S-transferase 2 | antimicrobial humoral response (sensu Vertebrata); cell-cell signaling; enzyme activator activity; glutathione transferase activity; integral to membrane; leukotriene biosynthesis; membrane fraction; microsome; signal transduction; transferase activity |
| J03143 | [IFNGR1](http://bioinfo.weizmann.ac.il/cards-bin/carddisp?IFNGR1) | interferon gamma receptor 1 | cytokine binding; hematopoietin/interferon-class (D200-domain) cytokine receptor activity; integral to plasma membrane; interferon-gamma receptor activity; receptor activity; response to pathogenic bacteria; response to virus; signal transduction |

Cluster P3

| ***GENBANK*** | ***SYMBOL*** | ***GENENAME*** | ***GENEONTOLOGY*** |
| --- | --- | --- | --- |
| U07418 | [MLH1](http://bioinfo.weizmann.ac.il/cards-bin/carddisp?MLH1) | mutL homolog 1, colon cancer, nonpolyposis type 2 (E. coli) | ATP binding; mismatch repair; negative regulation of cell cycle; nucleus |
| M29971 | [MGMT](http://bioinfo.weizmann.ac.il/cards-bin/carddisp?MGMT) | O-6-methylguanine-DNA methyltransferase | DNA binding; DNA ligation; DNA-methyltransferase activity; methylated-DNA-[protein]-cysteine S-methyltransferase activity; nucleus; transferase activity |
| U49844 | [ATR](http://bioinfo.weizmann.ac.il/cards-bin/carddisp?ATR) | ataxia telangiectasia and Rad3 related | DNA repair; cell cycle; cell cycle checkpoint; development; inositol/phosphatidylinositol kinase activity; protein kinase activity; transferase activity |
| NM_004862 | [LITAF](http://bioinfo.weizmann.ac.il/cards-bin/carddisp?LITAF) | lipopolysaccharide-induced TNF factor | RNA polymerase II transcription factor activity; nucleus; regulation of transcription from Pol II promoter |
| X04602 | [IL6](http://bioinfo.weizmann.ac.il/cards-bin/carddisp?IL6) | interleukin 6 (interferon, beta 2) | acute-phase response; cell proliferation; cell surface receptor linked signal transduction; cell-cell signaling; cytokine activity; development; extracellular space; humoral immune response; interleukin-6 receptor binding; negative regulation of cell proliferation; positive regulation of cell proliferation; skeletal development |
| U34605 | [IFIT5](http://bioinfo.weizmann.ac.il/cards-bin/carddisp?IFIT5) | interferon-induced protein with tetratricopeptide repeats 5 | biological_process unknown; cellular_component unknown; immune response; molecular_function unknown |
| X03557 | [IFIT1](http://bioinfo.weizmann.ac.il/cards-bin/carddisp?IFIT1) | interferon-induced protein with tetratricopeptide repeats 1 | biological_process unknown; cytoplasm; immune response; molecular_function unknown |
| X67325 | [IFI27](http://bioinfo.weizmann.ac.il/cards-bin/carddisp?IFI27) | interferon, alpha-inducible protein 27 | biological_process unknown; immune response; integral to membrane; molecular_function unknown |
| M80563 | [S100A4](http://bioinfo.weizmann.ac.il/cards-bin/carddisp?S100A4) | S100 calcium binding protein A4 (calcium protein, calvasculin, metastasin, murine placental homolog) | calcium ion binding |
| D86322 | [CLGN](http://bioinfo.weizmann.ac.il/cards-bin/carddisp?CLGN) | calmegin | calcium ion storage activity; chaperone activity; endoplasmic reticulum; fertilization (sensu Animalia); integral to membrane |
| Z18951 | [CAV1](http://bioinfo.weizmann.ac.il/cards-bin/carddisp?CAV1) | caveolin 1, caveolae protein, 22kDa | caveola; integral to plasma membrane; structural molecule activity |
| X03168 | [VTN](http://bioinfo.weizmann.ac.il/cards-bin/carddisp?VTN) | vitronectin (serum spreading factor, somatomedin B, complement S-protein) | cell adhesion; extracellular space; heparin binding; immune response; protein binding |
| J04164 | [IFITM1](http://bioinfo.weizmann.ac.il/cards-bin/carddisp?IFITM1) | interferon induced transmembrane protein 1 (9-27) | cell surface receptor linked signal transduction; immune response; integral to membrane; negative regulation of cell proliferation; plasma membrane; receptor signaling protein activity; regulation of cell cycle |
| L27943 | [CDA](http://bioinfo.weizmann.ac.il/cards-bin/carddisp?CDA) | cytidine deaminase | cytidine deaminase activity; cytidine metabolism; hydrolase activity; nucleobase, nucleoside, nucleotide and nucleic acid metabolism; zinc ion binding |
| Y15227 | [DLEU1](http://bioinfo.weizmann.ac.il/cards-bin/carddisp?DLEU1) | deleted in lymphocytic leukemia, 1 | negative regulation of cell cycle |
| Y08614 | [XPO1](http://bioinfo.weizmann.ac.il/cards-bin/carddisp?XPO1) | exportin 1 (CRM1 homolog, yeast) | protein-nucleus import; binding; nuclear pore; nucleoplasm; cytoplasm |
| X70904 | [LAMA4](http://bioinfo.weizmann.ac.il/cards-bin/carddisp?LAMA4) | laminin, alpha 4 | receptor binding; extracellular matrix structural constituent; protein binding; basal lamina; laminin-1 |

Cluster P4

| ***GENBANK*** | ***SYMBOL*** | ***GENENAME*** | ***GENEONTOLOGY*** |
| --- | --- | --- | --- |
| M74091 | [CCNC](http://bioinfo.weizmann.ac.il/cards-bin/carddisp?CCNC) | cyclin C | cytokinesis; nucleus; regulation of cell cycle; regulation of transcription, DNA-dependent |
| U76638 | [BARD1](http://bioinfo.weizmann.ac.il/cards-bin/carddisp?BARD1) | BRCA1 associated RING domain 1 | nucleus |
| X76104 | [DAPK1](http://bioinfo.weizmann.ac.il/cards-bin/carddisp?DAPK1) | death-associated protein kinase 1 | ATP binding; actin cytoskeleton; apoptosis; calcium/calmodulin-dependent protein kinase activity; calmodulin binding; calmodulin-dependent protein kinase I activity; induction of apoptosis by extracellular signals; protein amino acid phosphorylation; protein kinase cascade; protein serine/threonine kinase activity; signal transduction; transferase activity |
| M31899 | [ERCC3](http://bioinfo.weizmann.ac.il/cards-bin/carddisp?ERCC3) | excision repair cross-complementing rodent repair deficiency, complementation group 3 (xeroderma pigmentosum group B complementing) |  |
| NM_199187 | [KRT18](http://bioinfo.weizmann.ac.il/cards-bin/carddisp?KRT18) | keratin 18 |  |
| NM_000535 | [PMS2](http://bioinfo.weizmann.ac.il/cards-bin/carddisp?PMS2) | PMS2 postmeiotic segregation increased 2 (S. cerevisiae) | ATP binding; DNA binding; mismatch repair; negative regulation of cell cycle; nucleus |
| X57110 | [CBL](http://bioinfo.weizmann.ac.il/cards-bin/carddisp?CBL) | Cas-Br-M (murine) ecotropic retroviral transforming sequence | calcium ion binding; cell growth and/or maintenance; cell surface receptor linked signal transduction; ligase activity; nucleus; signal transducer activity; transcription factor activity |
| X57110 | CBL | Cas-Br-M (murine) ecotropic retroviral transforming sequence | calcium ion binding; cell growth and/or maintenance; cell surface receptor linked signal transduction; ligase activity; nucleus; signal transducer activity; transcription factor activity |
| L08246 | [MCL1](http://bioinfo.weizmann.ac.il/cards-bin/carddisp?MCL1) | myeloid cell leukemia sequence 1 (BCL2-related) | anti-apoptosis; apoptotic program; cell differentiation; cell fate determination; cell homeostasis; cytoplasm; integral to membrane; mitochondrial outer membrane; protein binding; protein channel activity; protein heterodimerization activity; regulation of apoptosis |
| X97795 | [RAD54L](http://bioinfo.weizmann.ac.il/cards-bin/carddisp?RAD54L) | RAD54-like (S. cerevisiae) |  |
| U33841 | [ATM](http://bioinfo.weizmann.ac.il/cards-bin/carddisp?ATM) | ataxia telangiectasia mutated (includes complementation groups A, C and D) | DNA binding; DNA repair; inositol/phosphatidylinositol kinase activity; meiotic recombination; negative regulation of cell cycle; nucleus; protein serine/threonine kinase activity; signal transduction; transferase activity |
| M97934 | [STAT2](http://bioinfo.weizmann.ac.il/cards-bin/carddisp?STAT2) | signal transducer and activator of transcription 2, 113kDa | JAK-STAT cascade; hematopoietin/interferon-class (D200-domain) cytokine receptor signal transducer activity; intracellular signaling cascade; nucleus; regulation of transcription from Pol II promoter; signal transducer activity; transcription factor activity |
| X80692 | [MAPK6](http://bioinfo.weizmann.ac.il/cards-bin/carddisp?MAPK6) | mitogen-activated protein kinase 6 | ATP binding; MAP kinase activity; cell cycle; protein amino acid phosphorylation; protein serine/threonine kinase activity; signal transduction; transferase activity |
| U21092 | [TRAF3](http://bioinfo.weizmann.ac.il/cards-bin/carddisp?TRAF3) | TNF receptor-associated factor 3 | apoptosis; induction of apoptosis; signal transducer activity; signal transduction; zinc ion binding |
| X02851 | [IL1A](http://bioinfo.weizmann.ac.il/cards-bin/carddisp?IL1A) | interleukin 1, alpha | anti-apoptosis; apoptosis; cell proliferation; cell-cell signaling; chemotaxis; cytoplasm; extracellular space; immune response; inflammatory response; interleukin-1 receptor binding; negative regulation of cell proliferation; regulation of cell cycle; signal transducer activity |
| Y00503 | [KRT19](http://bioinfo.weizmann.ac.il/cards-bin/carddisp?KRT19) | keratin 19 | intermediate filament; structural constituent of cytoskeleton |
| X06374 | [PDGFA](http://bioinfo.weizmann.ac.il/cards-bin/carddisp?PDGFA) | platelet-derived growth factor alpha polypeptide | cell proliferation; cell surface receptor linked signal transduction; cell-cell signaling; extracellular space; growth factor activity; membrane; platelet-derived growth factor receptor binding; regulation of cell cycle |
| X86779 | [FASTK](http://bioinfo.weizmann.ac.il/cards-bin/carddisp?FASTK) | FAST kinase | apoptosis; induction of apoptosis by extracellular signals; kinase activity; protein amino acid phosphorylation; protein serine/threonine kinase activity; signal transduction; transferase activity |
| NM_000586 | [IL2](http://bioinfo.weizmann.ac.il/cards-bin/carddisp?IL2) | interleukin 2 | T-cell differentiation; anti-apoptosis; antimicrobial humoral response (sensu Vertebrata); cell adhesion; cell growth and/or maintenance; cell-cell signaling; cytokine activity; extracellular space; immune response; interleukin-2 receptor binding; kinase activator activity; natural killer cell activation; positive regulation of cell growth; positive regulation of cell proliferation |
| D38551 | [RAD21](http://bioinfo.weizmann.ac.il/cards-bin/carddisp?RAD21) | RAD21 homolog (S. pombe) | apoptosis; cell cycle; chromosome segregation; double-strand break repair; meiotic recombination; mitosis; nucleus; protein binding |
| X65372 | [PTBP1](http://bioinfo.weizmann.ac.il/cards-bin/carddisp?PTBP1) | polypyrimidine tract binding protein 1 |  |
| U39657 | [MAP2K6](http://bioinfo.weizmann.ac.il/cards-bin/carddisp?MAP2K6) | mitogen-activated protein kinase kinase 6 | ATP binding; DNA damage induced protein phosphorylation; MAP kinase kinase activity; cell cycle arrest; protein serine/threonine kinase activity; protein-tyrosine kinase activity; signal transduction; transferase activity |
| M18112 | [ADPRT](http://bioinfo.weizmann.ac.il/cards-bin/carddisp?ADPRT) | ADP-ribosyltransferase (NAD+; poly (ADP-ribose) polymerase) |  |
| U60520 | [CASP8](http://bioinfo.weizmann.ac.il/cards-bin/carddisp?CASP8) | caspase 8, apoptosis-related cysteine protease | apoptotic program; caspase activity; cysteine-type peptidase activity; cytoskeleton; hydrolase activity; mitochondrion; protein binding; proteolysis and peptidolysis; regulation of apoptosis |
| U56390 | [CASP9](http://bioinfo.weizmann.ac.il/cards-bin/carddisp?CASP9) | caspase 9, apoptosis-related cysteine protease | apoptotic program; caspase activation via cytochrome c; caspase activity; enzyme activator activity; intracellular; protein binding; proteolysis and peptidolysis; regulation of apoptosis |
| M63488 | [RPA1](http://bioinfo.weizmann.ac.il/cards-bin/carddisp?RPA1) | replication protein A1, 70kDa | DNA recombination; DNA repair; DNA replication factor A complex; DNA-dependent DNA replication; nucleus; single-stranded DNA binding |
| U03506 | [SLC1A1](http://bioinfo.weizmann.ac.il/cards-bin/carddisp?SLC1A1) | solute carrier family 1 (neuronal/epithelial high affinity glutamate transporter, system Xag), member 1 | L-glutamate transport; L-glutamate transporter activity; dicarboxylic acid transport; integral to plasma membrane; membrane fraction; sodium:dicarboxylate symporter activity; symporter activity; synaptic transmission; transport |
| U09477 | [TP53BP1](http://bioinfo.weizmann.ac.il/cards-bin/carddisp?TP53BP1) | tumor protein p53 binding protein, 1 | P-element binding; cytoplasm; nucleoplasm; positive regulation of transcription, DNA-dependent; protein binding; signal transduction; transcriptional activator activity |
| AF015956 | [DAXX](http://bioinfo.weizmann.ac.il/cards-bin/carddisp?DAXX) | death-associated protein 6 | apoptosis; calcium ion binding; nucleus; regulation of transcription, DNA-dependent |
| U76248 | [SIAH2](http://bioinfo.weizmann.ac.il/cards-bin/carddisp?SIAH2) | seven in absentia homolog 2 (Drosophila) | cytoplasm; development; nucleus; small GTPase mediated signal transduction; transcription corepressor activity; ubiquitin-dependent protein catabolism |
| D14533 | [XPA](http://bioinfo.weizmann.ac.il/cards-bin/carddisp?XPA) | xeroderma pigmentosum, complementation group A | damaged DNA binding; nucleotide-excision repair; nucleus; protein binding |
| U83117 | [UBL1](http://bioinfo.weizmann.ac.il/cards-bin/carddisp?UBL1) | ubiquitin-like 1 (sentrin) | protein modification; ubiquitin cycle |

Cluster P5

| ***GENBANK*** | ***SYMBOL*** | ***GENENAME*** | ***GENEONTOLOGY*** |
| --- | --- | --- | --- |
| L33264 | [CDK10](http://bioinfo.weizmann.ac.il/cards-bin/carddisp?CDK10) | cyclin-dependent kinase (CDC2-like) 10 | ATP binding; cyclin-dependent protein kinase activity; kinase activity; negative regulation of cell proliferation; protein amino acid phosphorylation; protein serine/threonine kinase activity; transferase activity; traversing start control point of mitotic cell cycle |
| M15796 | [PCNA](http://bioinfo.weizmann.ac.il/cards-bin/carddisp?PCNA) | proliferating cell nuclear antigen | DNA binding; DNA polymerase processivity factor activity; DNA repair; DNA replication; cell proliferation; delta-DNA polymerase cofactor complex; nucleus; regulation of DNA replication; regulation of cell cycle |
| U65410 | [MAD2L1](http://bioinfo.weizmann.ac.il/cards-bin/carddisp?MAD2L1) | MAD2 mitotic arrest deficient-like 1 (yeast) | cell cycle; kinetochore; mitosis; mitotic checkpoint; nucleus |
| AF029082 | [SFN](http://bioinfo.weizmann.ac.il/cards-bin/carddisp?SFN) | stratifin | cell proliferation; cytoplasm; extracellular space; negative regulation of protein kinase activity; protein domain specific binding; protein kinase C inhibitor activity; regulation of cell cycle; signal transduction |
| U22897 | [NDP52](http://bioinfo.weizmann.ac.il/cards-bin/carddisp?NDP52) | nuclear domain 10 protein | Nucleus; viral life cycle |

Cluster P6

| ***GENBANK*** | ***SYMBOL*** | ***GENENAME*** | ***GENEONTOLOGY*** |
| --- | --- | --- | --- |
| X66365 | [CDK6](http://bioinfo.weizmann.ac.il/cards-bin/carddisp?CDK6) | cyclin-dependent kinase 6 | ATP binding; G1 phase of mitotic cell cycle; cytokinesis; protein amino acid phosphorylation; protein serine/threonine kinase activity; regulation of cell cycle; transferase activity |
| M14505 | [CDK4](http://bioinfo.weizmann.ac.il/cards-bin/carddisp?CDK4) | cyclin-dependent kinase 4 | ATP binding; G1/S transition of mitotic cell cycle; cyclin-dependent protein kinase activity; cytokinesis; protein amino acid phosphorylation; protein kinase activity; regulation of cell cycle; transferase activity |
| M68520 | [CDK2](http://bioinfo.weizmann.ac.il/cards-bin/carddisp?CDK2) | cyclin-dependent kinase 2 | ATP binding; G2/M transition of mitotic cell cycle; cell cycle; cyclin-dependent protein kinase activity; cytokinesis; cytoplasm; mitosis; nucleus; positive regulation of cell proliferation; protein amino acid phosphorylation; protein serine/threonine kinase activity; regulation of DNA replication; transferase activity; traversing start control point of mitotic cell cycle |
| X66364 | [CDK5](http://bioinfo.weizmann.ac.il/cards-bin/carddisp?CDK5) | cyclin-dependent kinase 5 | ATP binding; cell cycle; cyclin-dependent protein kinase activity; cytokinesis; protein amino acid phosphorylation; transferase activity |
| AF006484 | [CDK2AP1](http://bioinfo.weizmann.ac.il/cards-bin/carddisp?CDK2AP1) | CDK2-associated protein 1 | DNA binding; DNA-dependent DNA replication; S phase of mitotic cell cycle; cytoplasm; negative regulation of cell cycle; nucleus; protein amino acid phosphorylation; signal transducer activity |
| S85655 | [PHB](http://bioinfo.weizmann.ac.il/cards-bin/carddisp?PHB) | prohibitin | DNA metabolism; cell growth and/or maintenance |
| U28413 | [CKN1](http://bioinfo.weizmann.ac.il/cards-bin/carddisp?CKN1) | Cockayne syndrome 1 (classical) | DNA repair; RNA polymerase II transcription factor activity; nucleus; perception of sound; protein binding; regulation of transcription, DNA-dependent |
| U70310 | [FANCG](http://bioinfo.weizmann.ac.il/cards-bin/carddisp?FANCG) | Fanconi anemia, complementation group G | DNA repair; cell cycle checkpoint; damaged DNA binding; nucleus |
| U72649 | [BTG2](http://bioinfo.weizmann.ac.il/cards-bin/carddisp?BTG2) | BTG family, member 2 | DNA repair; negative regulation of cell proliferation; transcription factor activity |
| U19769 | [CENPF](http://bioinfo.weizmann.ac.il/cards-bin/carddisp?CENPF) | centromere protein F, 350/400ka (mitosin) | DNA replication and chromosome cycle; kinetochore; nucleus; regulation of mitosis; spindle |
| AF000546 | [P2RY5](http://bioinfo.weizmann.ac.il/cards-bin/carddisp?P2RY5) | purinergic receptor P2Y, G-protein coupled, 5 | G-protein coupled receptor protein signaling pathway; integral to membrane; purinergic nucleotide receptor activity, G-protein coupled; rhodopsin-like receptor activity |
| X59798 | [CCND1](http://bioinfo.weizmann.ac.il/cards-bin/carddisp?CCND1) | cyclin D1 (PRAD1: parathyroid adenomatosis 1) | G1/S transition of mitotic cell cycle; cell growth and/or maintenance; cellular_component unknown; cytokinesis; nucleus; regulation of cell cycle |
| U28413 | [FLJ12595](http://bioinfo.weizmann.ac.il/cards-bin/carddisp?FLJ12595) | hypothetical protein FLJ12595 | GTP binding |
| Y07923 | [RND1](http://bioinfo.weizmann.ac.il/cards-bin/carddisp?RND1) | Rho family GTPase 1 | GTP binding; Rho small monomeric GTPase activity; actin filament organization; adherens junction; cytoskeleton; negative regulation of cell adhesion; neuronal remodeling; small GTPase mediated signal transduction |
| M33195 | [FCER1G](http://bioinfo.weizmann.ac.il/cards-bin/carddisp?FCER1G) | Fc fragment of IgE, high affinity I, receptor for; gamma polypeptide | IgE binding; cell surface receptor linked signal transduction; immune response; integral to plasma membrane; receptor signaling protein activity; transmembrane receptor activity |
| U77493 | [NOTCH2](http://bioinfo.weizmann.ac.il/cards-bin/carddisp?NOTCH2) | Notch homolog 2 (Drosophila) | Notch signaling pathway; anti-apoptosis; calcium ion binding; cell cycle arrest; cell differentiation; cell fate determination; cell growth; cell surface; determination of left/right symmetry; hemopoiesis; induction of apoptosis; integral to plasma membrane; ligand-regulated transcription factor activity; morphogenesis of an epithelial sheet; negative regulation of cell proliferation; neurogenesis; nucleus; positive regulation of RAS protein signal transduction; protein binding; protein heterodimerization activity; receptor activity; regulation of development; regulation of transcription, DNA-dependent; stem cell maintenance; transcription regulator activity |
| U43899 | [STAM](http://bioinfo.weizmann.ac.il/cards-bin/carddisp?STAM) | signal transducing adaptor molecule (SH3 domain and ITAM motif) 1 | SH3/SH2 adaptor protein activity; intracellular protein transport; signal transduction |
| AF010309 | [TP53I3](http://bioinfo.weizmann.ac.il/cards-bin/carddisp?TP53I3) | tumor protein p53 inducible protein 3 | alcohol dehydrogenase activity, zinc-dependent; biological_process unknown; cellular_component unknown; induction of apoptosis by oxidative stress; molecular_function unknown; zinc ion binding |
| U00001 | [CDC27](http://bioinfo.weizmann.ac.il/cards-bin/carddisp?CDC27) | cell division cycle 27 | anaphase-promoting complex; cell proliferation; centrosome; mitotic metaphase/anaphase transition; nucleus; spindle |
| U90313 | [GSTO1](http://bioinfo.weizmann.ac.il/cards-bin/carddisp?GSTO1) | glutathione S-transferase omega 1 | biological_process unknown; cytoplasm; glutathione transferase activity; metabolism; monodehydroascorbate reductase (NADH) activity; transferase activity |
| M55172 | [AGC1](http://bioinfo.weizmann.ac.il/cards-bin/carddisp?AGC1) | aggrecan 1 (chondroitin sulfate proteoglycan 1, large aggregating proteoglycan, antigen identified by monoclonal antibody A0122) | cell adhesion; extracellular matrix; heterophilic cell adhesion; hyaluronic acid binding; sugar binding |
| U59435 | [PA2G4](http://bioinfo.weizmann.ac.il/cards-bin/carddisp?PA2G4) | proliferation-associated 2G4, 38kDa | cell cycle arrest; cell proliferation; hydrolase activity; methionyl aminopeptidase activity; nucleus; proteolysis and peptidolysis |
| X74262 | [RBBP4](http://bioinfo.weizmann.ac.il/cards-bin/carddisp?RBBP4) | retinoblastoma binding protein 4 | Nucleus; DNA replication; DNA repair; transcription; regulation of transcription |

Cluster P7

| ***GENBANK*** | ***SYMBOL*** | ***GENENAME*** | ***GENEONTOLOGY*** |
| --- | --- | --- | --- |
| X52142 | [CTPS](http://bioinfo.weizmann.ac.il/cards-bin/carddisp?CTPS) | CTP synthase | CTP synthase activity; glutamine metabolism; ligase activity; nucleobase, nucleoside, nucleotide and nucleic acid metabolism; pyrimidine nucleotide biosynthesis; response to drug |
| X59618 | [RRM2](http://bioinfo.weizmann.ac.il/cards-bin/carddisp?RRM2) | ribonucleotide reductase M2 polypeptide | DNA replication; cytoplasm; deoxyribonucleoside diphosphate metabolism; oxidoreductase activity; ribonucleoside-diphosphate reductase activity |
| NM_001168 | [BIRC5](http://bioinfo.weizmann.ac.il/cards-bin/carddisp?BIRC5) | baculoviral IAP repeat-containing 5 (survivin) | G2/M transition of mitotic cell cycle; anti-apoptosis; apoptosis; cysteine protease inhibitor activity; intracellular; spindle microtubule |
| U27768 | [RGS4](http://bioinfo.weizmann.ac.il/cards-bin/carddisp?RGS4) | regulator of G-protein signalling 4 | GTPase activator activity; calmodulin binding; inactivation of MAPK; regulation of G-protein coupled receptor protein signaling pathway; signal transducer activity; signal transduction |
| X62744 | [HLA-DMA](http://bioinfo.weizmann.ac.il/cards-bin/carddisp?HLA-DMA) | major histocompatibility complex, class II, DM alpha | MHC class II receptor activity; antigen presentation, exogenous antigen; antigen processing, exogenous antigen via MHC class II; chaperone activity; detection of pest/pathogen/parasite; immune response; integral to membrane; protein binding |
| D13365 | [MT3](http://bioinfo.weizmann.ac.il/cards-bin/carddisp?MT3) | metallothionein 3 (growth inhibitory factor (neurotrophic)) | antioxidant activity; cell proliferation; copper ion binding; electron transport; electron transporter activity; metal ion binding; metal ion homeostasis; negative regulation of cell growth; negative regulation of dendrite morphogenesis; removal of superoxide radicals; response to hypoxia; synaptic vesicle; zinc ion binding |
| U18321 | [DAP3](http://bioinfo.weizmann.ac.il/cards-bin/carddisp?DAP3) | death associated protein 3 | apoptosis; induction of apoptosis by extracellular signals; mitochondrial ribosome; mitochondrion; small ribosomal subunit; structural constituent of ribosome |
| X07819 | [MMP7](http://bioinfo.weizmann.ac.il/cards-bin/carddisp?MMP7) | matrix metalloproteinase 7 (matrilysin, uterine) | calcium ion binding; collagen catabolism; extracellular matrix; extracellular space; hydrolase activity; matrilysin activity; zinc ion binding |
| X57766 | [MMP11](http://bioinfo.weizmann.ac.il/cards-bin/carddisp?MMP11) | matrix metalloproteinase 11 (stromelysin 3) | calcium ion binding; collagen catabolism; extracellular matrix; hydrolase activity; morphogenesis; stromelysin 3 activity; zinc ion binding |
| NM_001168 | [EPR1](http://bioinfo.weizmann.ac.il/cards-bin/carddisp?EPR1) | effector cell protease receptor 1 | cell surface receptor linked signal transduction; integral to membrane; peptidase activity; receptor activity |
| NM_001907 | [CTRL](http://bioinfo.weizmann.ac.il/cards-bin/carddisp?CTRL) | chymotrypsin-like | chymotrypsin activity; digestion; extracellular space; hydrolase activity; peptidase activity; proteolysis and peptidolysis; trypsin activity |
| X01677 | [GAPD](http://bioinfo.weizmann.ac.il/cards-bin/carddisp?GAPD) | glyceraldehyde-3-phosphate dehydrogenase | cytoplasm; glucose metabolism; glyceraldehyde-3-phosphate dehydrogenase (phosphorylating) activity; glycolysis; oxidoreductase activity |
| X56134 | [VIM](http://bioinfo.weizmann.ac.il/cards-bin/carddisp?VIM) | vimentin | cytoplasm; intermediate filament; protein binding; structural constituent of cytoskeleton |
| X03124 | [TIMP1](http://bioinfo.weizmann.ac.il/cards-bin/carddisp?TIMP1) | tissue inhibitor of metalloproteinase 1 (erythroid potentiating activity, collagenase inhibitor) | development; extracellular matrix; metalloendopeptidase inhibitor activity; metallopeptidase activity; positive regulation of cell proliferation; proteolysis and peptidolysis |
| X91911 | [GLIPR1](http://bioinfo.weizmann.ac.il/cards-bin/carddisp?GLIPR1) | GLI pathogenesis-related 1 (glioma) | extracellular |
| X92106 | [BLMH](http://bioinfo.weizmann.ac.il/cards-bin/carddisp?BLMH) | bleomycin hydrolase | aminopeptidase activity; carboxypeptidase activity; nucleus; cytoplasm; proteolysis and peptidolysis |
| X91247 | [TXNRD1](http://bioinfo.weizmann.ac.il/cards-bin/carddisp?TXNRD1) | thioredoxin reductase 1 | thioredoxin-disulfide reductase activity; cytoplasm; electron transport; signal transduction; disulfide oxidoreductase activity |

SISgel

Cluster S1

| ***GENBANK*** | ***SYMBOL*** | ***GENENAME*** | ***GENEONTOLOGY*** |
| --- | --- | --- | --- |
| X80692 | [MAPK6](http://bioinfo.weizmann.ac.il/cards-bin/carddisp?MAPK6) | mitogen-activated protein kinase 6 | ATP binding; MAP kinase activity; cell cycle; protein amino acid phosphorylation; protein serine/threonine kinase activity; signal transduction; transferase activity |
| M11886 | [HLA-C](http://bioinfo.weizmann.ac.il/cards-bin/carddisp?HLA-C) | major histocompatibility complex, class I, C | MHC class I receptor activity; MHC class II receptor activity; antigen presentation, endogenous antigen; antigen processing, endogenous antigen via MHC class I; immune response; integral to membrane |
| K02770 | [IL1B](http://bioinfo.weizmann.ac.il/cards-bin/carddisp?IL1B) | interleukin 1, beta | antimicrobial humoral response (sensu Vertebrata); apoptosis; cell proliferation; cell-cell signaling; extracellular space; immune response; inflammatory response; interleukin-1 receptor binding; negative regulation of cell proliferation; regulation of cell cycle; signal transducer activity; signal transduction |
| U56390 | [CASP9](http://bioinfo.weizmann.ac.il/cards-bin/carddisp?CASP9) | caspase 9, apoptosis-related cysteine protease | apoptotic program; caspase activation via cytochrome c; caspase activity; enzyme activator activity; intracellular; protein binding; proteolysis and peptidolysis; regulation of apoptosis |
| X57766 | [MMP11](http://bioinfo.weizmann.ac.il/cards-bin/carddisp?MMP11) | matrix metalloproteinase 11 (stromelysin 3) | calcium ion binding; collagen catabolism; extracellular matrix; hydrolase activity; morphogenesis; stromelysin 3 activity; zinc ion binding |
| U60519 | [CASP10](http://bioinfo.weizmann.ac.il/cards-bin/carddisp?CASP10) | caspase 10, apoptosis-related cysteine protease | caspase activity; cysteine-type peptidase activity; hydrolase activity; induction of apoptosis; peptidase activity; protein binding; proteolysis and peptidolysis; regulation of apoptosis |
| X03168 | [VTN](http://bioinfo.weizmann.ac.il/cards-bin/carddisp?VTN) | vitronectin (serum spreading factor, somatomedin B, complement S-protein) | cell adhesion; extracellular space; heparin binding; immune response; protein binding |
| U09579 | [CDKN1A](http://bioinfo.weizmann.ac.il/cards-bin/carddisp?CDKN1A) | cyclin-dependent kinase inhibitor 1A (p21, Cip1) | cell cycle arrest; cyclin-dependent protein kinase inhibitor activity; induction of apoptosis by intracellular signals; kinase activity; negative regulation of cell proliferation; nucleus; protein kinase activity; regulation of CDK activity |
| X02811 | [PDGFB](http://bioinfo.weizmann.ac.il/cards-bin/carddisp?PDGFB) | platelet-derived growth factor beta polypeptide (simian sarcoma viral (v-sis) oncogene homolog) | cell proliferation; extracellular; growth factor activity; membrane; platelet-derived growth factor receptor binding; regulation of cell cycle; response to wounding |
| X03124 | [TIMP1](http://bioinfo.weizmann.ac.il/cards-bin/carddisp?TIMP1) | tissue inhibitor of metalloproteinase 1 (erythroid potentiating activity, collagenase inhibitor) | development; extracellular matrix; metalloendopeptidase inhibitor activity; metallopeptidase activity; positive regulation of cell proliferation; proteolysis and peptidolysis |
| X89576 | [MMP17](http://bioinfo.weizmann.ac.il/cards-bin/carddisp?MMP17) | matrix metalloproteinase 17 (membrane-inserted) | enzyme activator activity; extracellular matrix; hydrolase activity; integral to plasma membrane; metalloendopeptidase activity; proteolysis and peptidolysis; zinc ion binding |
| X52773 | [RXRA](http://bioinfo.weizmann.ac.il/cards-bin/carddisp?RXRA) | retinoid X receptor, alpha | nucleus; receptor activity; regulation of transcription, DNA-dependent; retinoid-X receptor activity; signal transduction; steroid binding; steroid hormone receptor activity; transcription coactivator activity; transcription factor activity; vitamin metabolism |
| U05875 | [IFNGR2](http://bioinfo.weizmann.ac.il/cards-bin/carddisp?IFNGR2) | interferon gamma receptor 2 (interferon gamma transducer 1) | receptor activity; hematopoietin/interferon-class (D200-domain) cytokine receptor activity; interferon-gamma receptor activity; integral to plasma membrane; cell surface receptor linked signal transduction |
| U14971 | [RPS9](http://bioinfo.weizmann.ac.il/cards-bin/carddisp?RPS9) | ribosomal protein S9 | RNA binding; structural constituent of ribosome; intracellular; cytosolic small ribosomal subunit (sensu Eukaryota); protein biosynthesis |

Cluster S2

| ***GENBANK*** | ***SYMBOL*** | ***GENENAME*** | ***GENEONTOLOGY*** |
| --- | --- | --- | --- |
| X60188 | [MAPK3](http://bioinfo.weizmann.ac.il/cards-bin/carddisp?MAPK3) | mitogen-activated protein kinase 3 | ATP binding; MAP kinase activity; cellular_component unknown; protein amino acid phosphorylation; protein serine/threonine kinase activity; regulation of cell cycle; transferase activity |
| M57627 | [IL10](http://bioinfo.weizmann.ac.il/cards-bin/carddisp?IL10) | interleukin 10 | B-cell differentiation; B-cell proliferation; T-helper 2 type immune response; anti-apoptosis; cell-cell signaling; cytokine activity; cytoplasmic sequestering of NF-kappaB; extracellular; hemopoiesis; immune cell chemotaxis; immune response; interleukin-10 receptor binding; negative regulation of MHC class II biosynthesis; negative regulation of T-cell proliferation; negative regulation of interferon-alpha biosynthesis; negative regulation of interferon-gamma biosynthesis; negative regulation of nitric oxide biosynthesis; regulation of isotype switching |
| M28622 | [IFNB1](http://bioinfo.weizmann.ac.il/cards-bin/carddisp?IFNB1) | interferon, beta 1, fibroblast | B-cell proliferation; anti-inflammatory response; caspase activation; cell surface receptor linked signal transduction; defense response; extracellular; interferon-alpha/beta receptor binding; natural killer cell activation; negative regulation of cell proliferation; negative regulation of virion penetration; positive regulation of innate immune response; regulation of MHC class I biosynthesis; response to virus |
| U12255 | [FCGRT](http://bioinfo.weizmann.ac.il/cards-bin/carddisp?FCGRT) | Fc fragment of IgG, receptor, transporter, alpha | IgG binding; immune response; integral to membrane; pregnancy; receptor activity |
| X74295 | [MGC17301](http://bioinfo.weizmann.ac.il/cards-bin/carddisp?MGC17301) | hypothetical protein MGC17301 | S-adenosylmethionine-dependent methyltransferase activity |
| NM_000586 | [IL2](http://bioinfo.weizmann.ac.il/cards-bin/carddisp?IL2) | interleukin 2 | T-cell differentiation; anti-apoptosis; antimicrobial humoral response (sensu Vertebrata); cell adhesion; cell growth and/or maintenance; cell-cell signaling; cytokine activity; extracellular space; immune response; interleukin-2 receptor binding; kinase activator activity; natural killer cell activation; positive regulation of cell growth; positive regulation of cell proliferation |
| X02851 | [IL1A](http://bioinfo.weizmann.ac.il/cards-bin/carddisp?IL1A) | interleukin 1, alpha | anti-apoptosis; apoptosis; cell proliferation; cell-cell signaling; chemotaxis; cytoplasm; extracellular space; immune response; inflammatory response; interleukin-1 receptor binding; negative regulation of cell proliferation; regulation of cell cycle; signal transducer activity |
| L08096 | [TNFSF7](http://bioinfo.weizmann.ac.il/cards-bin/carddisp?TNFSF7) | tumor necrosis factor (ligand) superfamily, member 7 | apoptosis; cell proliferation; cell-cell signaling; immune response; integral to plasma membrane; signal transduction; tumor necrosis factor receptor binding |
| U09304 | [EFNB1](http://bioinfo.weizmann.ac.il/cards-bin/carddisp?EFNB1) | ephrin-B1 | cell adhesion; cell-cell signaling; development; ephrin receptor binding; integral to plasma membrane; neurogenesis; soluble fraction |
| M55172 | [AGC1](http://bioinfo.weizmann.ac.il/cards-bin/carddisp?AGC1) | aggrecan 1 (chondroitin sulfate proteoglycan 1, large aggregating proteoglycan, antigen identified by monoclonal antibody A0122) | cell adhesion; extracellular matrix; heterophilic cell adhesion; hyaluronic acid binding; sugar binding |
| X06374 | [PDGFA](http://bioinfo.weizmann.ac.il/cards-bin/carddisp?PDGFA) | platelet-derived growth factor alpha polypeptide | cell proliferation; cell surface receptor linked signal transduction; cell-cell signaling; extracellular space; growth factor activity; membrane; platelet-derived growth factor receptor binding; regulation of cell cycle |
| M54995 | [PPBP](http://bioinfo.weizmann.ac.il/cards-bin/carddisp?PPBP) | pro-platelet basic protein (chemokine (C-X-C motif) ligand 7) | cell proliferation; chemokine activity; chemotaxis; extracellular; extracellular matrix structural constituent; glucose transport; glucose transporter activity; growth factor activity; immune response; regulation of cell cycle |
| X74295 | [ITGA7](http://bioinfo.weizmann.ac.il/cards-bin/carddisp?ITGA7) | integrin, alpha 7 | cell-matrix adhesion; cellular morphogenesis; homophilic cell adhesion; integrin complex; integrin-mediated signaling pathway; muscle development; protein binding; receptor activity |
| NM_002171 | [IFNA10](http://bioinfo.weizmann.ac.il/cards-bin/carddisp?IFNA10) | interferon, alpha 10 | defense response; extracellular; interferon-alpha/beta receptor binding; response to virus |
| U49262 | [DVL3](http://bioinfo.weizmann.ac.il/cards-bin/carddisp?DVL3) | dishevelled, dsh homolog 3 (Drosophila) | development; frizzled signaling pathway; heart development; intracellular; intracellular signaling cascade; kinase activity; neurogenesis; protein binding; signal transducer activity |

Cluster S3

| ***GENBANK*** | ***SYMBOL*** | ***GENENAME*** | ***GENEONTOLOGY*** |
| --- | --- | --- | --- |
| M63167 | [AKT1](http://bioinfo.weizmann.ac.il/cards-bin/carddisp?AKT1) | v-akt murine thymoma viral oncogene homolog 1 | ATP binding; G-protein coupled receptor protein signaling pathway; anti-apoptosis; nitric oxide biosynthesis; nucleus; protein amino acid phosphorylation; receptor signaling protein serine/threonine kinase activity; response to heat; signal transduction; transferase activity |
| X76104 | [DAPK1](http://bioinfo.weizmann.ac.il/cards-bin/carddisp?DAPK1) | death-associated protein kinase 1 | ATP binding; actin cytoskeleton; apoptosis; calcium/calmodulin-dependent protein kinase activity; calmodulin binding; calmodulin-dependent protein kinase I activity; induction of apoptosis by extracellular signals; protein amino acid phosphorylation; protein kinase cascade; protein serine/threonine kinase activity; signal transduction; transferase activity |
| M32865 | [G22P1](http://bioinfo.weizmann.ac.il/cards-bin/carddisp?G22P1) | thyroid autoantigen 70kDa (Ku antigen) | ATP-dependent DNA helicase activity; DNA ligation; double-strand break repair via nonhomologous end-joining; double-stranded DNA binding; helicase activity; membrane fraction; nucleus |
| S74678 | [HNRPK](http://bioinfo.weizmann.ac.il/cards-bin/carddisp?HNRPK) | heterogeneous nuclear ribonucleoprotein K | DNA binding; RNA binding; RNA processing; heterogeneous nuclear ribonucleoprotein complex; nucleoplasm |
| M15024 | [MYB](http://bioinfo.weizmann.ac.il/cards-bin/carddisp?MYB) | v-myb myeloblastosis viral oncogene homolog (avian) | DNA binding; cell growth and/or maintenance; nuclear matrix; nucleus; regulation of transcription, DNA-dependent; transcriptional activator activity |
| NM_003449 | [TRIM26](http://bioinfo.weizmann.ac.il/cards-bin/carddisp?TRIM26) | tripartite motif-containing 26 | DNA binding; intracellular; protein binding; zinc ion binding |
| S85655 | [PHB](http://bioinfo.weizmann.ac.il/cards-bin/carddisp?PHB) | prohibitin | DNA metabolism; cell growth and/or maintenance |
| X59543 | [RRM1](http://bioinfo.weizmann.ac.il/cards-bin/carddisp?RRM1) | ribonucleotide reductase M1 polypeptide | DNA replication; oxidoreductase activity; ribonucleoside-diphosphate reductase activity; ribonucleoside-diphosphate reductase complex |
| U22398 | [CDKN1C](http://bioinfo.weizmann.ac.il/cards-bin/carddisp?CDKN1C) | cyclin-dependent kinase inhibitor 1C (p57, Kip2) | G1 phase of mitotic cell cycle; cell cycle; cell cycle arrest; cyclin-dependent protein kinase inhibitor activity; negative regulation of cell cycle; negative regulation of cell proliferation; nucleus; regulation of CDK activity |
| M61764 | [TUBG1](http://bioinfo.weizmann.ac.il/cards-bin/carddisp?TUBG1) | tubulin, gamma 1 | GTP binding; centrosome; microtubule; microtubule cytoskeleton organization and biogenesis; microtubule-based movement; spindle pole body; structural constituent of cytoskeleton |
| U37436 | [ATIC](http://bioinfo.weizmann.ac.il/cards-bin/carddisp?ATIC) | 5-aminoimidazole-4-carboxamide ribonucleotide formyltransferase/IMP cyclohydrolase | IMP cyclohydrolase activity; hydrolase activity; nucleobase, nucleoside, nucleotide and nucleic acid metabolism; phosphoribosylaminoimidazolecarboxamide formyltransferase activity; purine nucleotide biosynthesis; transferase activity |
| X87838 | [CTNNB1](http://bioinfo.weizmann.ac.il/cards-bin/carddisp?CTNNB1) | catenin (cadherin-associated protein), beta 1, 88kDa | Wnt receptor signaling pathway; cell adhesion; cytoskeleton; intercellular junction; nucleus; plasma membrane; protein binding; regulation of transcription from Pol II promoter; signal transducer activity; structural molecule activity; transcription |
| X02812 | [TGFB1](http://bioinfo.weizmann.ac.il/cards-bin/carddisp?TGFB1) | transforming growth factor, beta 1 (Camurati-Engelmann disease) | anti-apoptosis; cell growth; cell proliferation; cell-cell signaling; growth; regulation of cell cycle; transforming growth factor beta receptor binding; transforming growth factor beta receptor signaling pathway |
| X86779 | [FASTK](http://bioinfo.weizmann.ac.il/cards-bin/carddisp?FASTK) | FAST kinase | apoptosis; induction of apoptosis by extracellular signals; kinase activity; protein amino acid phosphorylation; protein serine/threonine kinase activity; signal transduction; transferase activity |
| U21092 | [TRAF3](http://bioinfo.weizmann.ac.il/cards-bin/carddisp?TRAF3) | TNF receptor-associated factor 3 | apoptosis; induction of apoptosis; signal transducer activity; signal transduction; zinc ion binding |
| U69108 | [TRAF5](http://bioinfo.weizmann.ac.il/cards-bin/carddisp?TRAF5) | TNF receptor-associated factor 5 | apoptosis; signal transduction; zinc ion binding |
| D13866 | [CTNNA1](http://bioinfo.weizmann.ac.il/cards-bin/carddisp?CTNNA1) | catenin (cadherin-associated protein), alpha 1, 102kDa | cell adhesion; cytoskeleton; protein binding; structural molecule activity |
| M30938 | [XRCC5](http://bioinfo.weizmann.ac.il/cards-bin/carddisp?XRCC5) | X-ray repair complementing defective repair in Chinese hamster cells 5 (double-strand-break rejoining; Ku autoantigen, 80kDa) | cellular_component unknown; molecular_function unknown; regulation of DNA repair |
| M34225 | [KRT8](http://bioinfo.weizmann.ac.il/cards-bin/carddisp?KRT8) | keratin 8 | cytoskeleton organization and biogenesis; intermediate filament; phosphorylation; structural molecule activity |
| U10860 | [GMPS](http://bioinfo.weizmann.ac.il/cards-bin/carddisp?GMPS) | guanine monphosphate synthetase | GMP synthase activity; GMP synthase (glutamine-hydrolyzing) activity; anthranilate synthase activity; ATP binding; purine nucleotide biosynthesis |
| NM_199187 | [KRT18](http://bioinfo.weizmann.ac.il/cards-bin/carddisp?KRT18) | keratin 18 | structural constituent of cytoskeleton; intermediate filament; morphogenesis |
| J04031 | [MTHFD1](http://bioinfo.weizmann.ac.il/cards-bin/carddisp?MTHFD1) | methylenetetrahydrofolate dehydrogenase (NADP+ dependent), methenyltetrahydrofolate cyclohydrolase, formyltetrahydrofolate synthetase | histidine biosynthesis; formate-tetrahydrofolate ligase activity; methenyltetrahydrofolate cyclohydrolase activity; methylenetetrahydrofolate dehydrogenase (NADP+) activity; ATP binding |
| X91247 | [TXNRD1](http://bioinfo.weizmann.ac.il/cards-bin/carddisp?TXNRD1) | thioredoxin reductase 1 | thioredoxin-disulfide reductase activity; cytoplasm; electron transport; signal transduction; disulfide oxidoreductase activity |
| J03250 | [TOP1](http://bioinfo.weizmann.ac.il/cards-bin/carddisp?TOP1) | topoisomerase (DNA) I | DNA topoisomerase type I activity; DNA topological change; DNA unwinding |
| J04088 | [TOP2A](http://bioinfo.weizmann.ac.il/cards-bin/carddisp?TOP2A) | topoisomerase (DNA) II alpha 170kDa | DNA topoisomerase (ATP-hydrolyzing) activity; ATP binding; nucleus; DNA topological change |

Cluster S4

| ***GENBANK*** | ***SYMBOL*** | ***GENENAME*** | ***GENEONTOLOGY*** |
| --- | --- | --- | --- |
| NM_000535 | [PMS2](http://bioinfo.weizmann.ac.il/cards-bin/carddisp?PMS2) | PMS2 postmeiotic segregation increased 2 (S. cerevisiae) | ATP binding; DNA binding; mismatch repair; negative regulation of cell cycle; nucleus |
| U69127 | [FUBP3](http://bioinfo.weizmann.ac.il/cards-bin/carddisp?FUBP3) | far upstream element (FUSE) binding protein 3 | DNA binding; nucleus; regulation of transcription, DNA-dependent |
| NM_001118 | [ADCYAP1R1](http://bioinfo.weizmann.ac.il/cards-bin/carddisp?ADCYAP1R1) | adenylate cyclase activating polypeptide 1 (pituitary) receptor type I | G-protein coupled receptor activity; G-protein coupled receptor protein signaling pathway; integral to plasma membrane; receptor activity; spermatogenesis; vasoactive intestinal polypeptide receptor activity |
| M35416 | [RALB](http://bioinfo.weizmann.ac.il/cards-bin/carddisp?RALB) | v-ral simian leukemia viral oncogene homolog B (ras related; GTP binding protein) | GTP binding; signal transduction; small GTPase mediated signal transduction; small monomeric GTPase activity |
| M97934 | [STAT2](http://bioinfo.weizmann.ac.il/cards-bin/carddisp?STAT2) | signal transducer and activator of transcription 2, 113kDa | JAK-STAT cascade; hematopoietin/interferon-class (D200-domain) cytokine receptor signal transducer activity; intracellular signaling cascade; nucleus; regulation of transcription from Pol II promoter; signal transducer activity; transcription factor activity |
| U77604 | [MGST2](http://bioinfo.weizmann.ac.il/cards-bin/carddisp?MGST2) | microsomal glutathione S-transferase 2 | antimicrobial humoral response (sensu Vertebrata); cell-cell signaling; enzyme activator activity; glutathione transferase activity; integral to membrane; leukotriene biosynthesis; membrane fraction; microsome; signal transduction; transferase activity |
| U60520 | [CASP8](http://bioinfo.weizmann.ac.il/cards-bin/carddisp?CASP8) | caspase 8, apoptosis-related cysteine protease | apoptotic program; caspase activity; cysteine-type peptidase activity; cytoskeleton; hydrolase activity; mitochondrion; protein binding; proteolysis and peptidolysis; regulation of apoptosis |
| U60062 | [FEZ1](http://bioinfo.weizmann.ac.il/cards-bin/carddisp?FEZ1) | fasciculation and elongation protein zeta 1 (zygin I) | axon guidance; cell adhesion; neurogenesis |
| Z18951 | [CAV1](http://bioinfo.weizmann.ac.il/cards-bin/carddisp?CAV1) | caveolin 1, caveolae protein, 22kDa | caveola; integral to plasma membrane; structural molecule activity |
| X15879 | [COL6A1](http://bioinfo.weizmann.ac.il/cards-bin/carddisp?COL6A1) | collagen, type VI, alpha 1 | cell adhesion; collagen type VI; extracellular matrix; extracellular matrix structural constituent; molecular_function unknown; protein binding |
| X91940 | [WNT8B](http://bioinfo.weizmann.ac.il/cards-bin/carddisp?WNT8B) | wingless-type MMTV integration site family, member 8B | cell-cell signaling; development; extracellular; frizzled-2 signaling pathway; neurogenesis; signal transducer activity; signal transduction |
| X59131 | [D13S106E](http://bioinfo.weizmann.ac.il/cards-bin/carddisp?D13S106E) | highly charged protein | cysteine-type endopeptidase activity; ubiquitin thiolesterase activity; ubiquitin-dependent protein catabolism |
| L27943 | [CDA](http://bioinfo.weizmann.ac.il/cards-bin/carddisp?CDA) | cytidine deaminase | cytidine deaminase activity; cytidine metabolism; hydrolase activity; nucleobase, nucleoside, nucleotide and nucleic acid metabolism; zinc ion binding |
| NM_001233 | [CAV2](http://bioinfo.weizmann.ac.il/cards-bin/carddisp?CAV2) | caveolin 2 | integral to membrane |
| M82882 | [ELF1](http://bioinfo.weizmann.ac.il/cards-bin/carddisp?ELF1) | E74-like factor 1 (ets domain transcription factor) | nucleus; regulation of transcription, DNA-dependent; transcription factor activity; transcriptional activator activity |
| X89059 | [CDKL5](http://bioinfo.weizmann.ac.il/cards-bin/carddisp?CDKL5) | cyclin-dependent kinase-like 5 | protein serine/threonine kinase activity; ATP binding; protein amino acid phosphorylation; transferase activity |
| NM_003544 | [HIST1H4B](http://bioinfo.weizmann.ac.il/cards-bin/carddisp?HIST1H4B) | histone 1, H4b | Nucleosome; DNA binding; nucleus; chromosome; DNA packaging |
| U14966 | [RPL5](http://bioinfo.weizmann.ac.il/cards-bin/carddisp?RPL5) | ribosomal protein L5 | structural constituent of ribosome; intracellular; ribosome; cytosolic large ribosomal subunit (sensu Eukaryota); protein biosynthesis |

Cluster S5

| ***GENBANK*** | ***SYMBOL*** | ***GENENAME*** | ***GENEONTOLOGY*** |
| --- | --- | --- | --- |
| NM_000534 | [PMS1](http://bioinfo.weizmann.ac.il/cards-bin/carddisp?PMS1) | PMS1 postmeiotic segregation increased 1 (S. cerevisiae) | ATP binding; DNA binding; mismatch repair; negative regulation of cell cycle; nucleus; regulation of transcription, DNA-dependent |
| U07418 | [MLH1](http://bioinfo.weizmann.ac.il/cards-bin/carddisp?MLH1) | mutL homolog 1, colon cancer, nonpolyposis type 2 (E. coli) | ATP binding; mismatch repair; negative regulation of cell cycle; nucleus |
| L25081 | [ARHC](http://bioinfo.weizmann.ac.il/cards-bin/carddisp?ARHC) | ras homolog gene family, member C | GTP binding; Rho small monomeric GTPase activity; catalytic activity; cell growth and/or maintenance; small GTPase mediated signal transduction |
| X67325 | [IFI27](http://bioinfo.weizmann.ac.il/cards-bin/carddisp?IFI27) | interferon, alpha-inducible protein 27 | biological_process unknown; immune response; integral to membrane; molecular_function unknown |
| NM_002203 | [ITGA2](http://bioinfo.weizmann.ac.il/cards-bin/carddisp?ITGA2) | integrin, alpha 2 (CD49B, alpha 2 subunit of VLA-2 receptor) | blood coagulation; cell-matrix adhesion; collagen binding; integral to membrane; integrin complex; integrin-mediated signaling pathway; magnesium ion binding; organogenesis; plasma membrane; receptor activity |
| U53446 | [DAB2](http://bioinfo.weizmann.ac.il/cards-bin/carddisp?DAB2) | disabled homolog 2, mitogen-responsive phosphoprotein (Drosophila) | cell proliferation |
| J04164 | [IFITM1](http://bioinfo.weizmann.ac.il/cards-bin/carddisp?IFITM1) | interferon induced transmembrane protein 1 (9-27) | cell surface receptor linked signal transduction; immune response; integral to membrane; negative regulation of cell proliferation; plasma membrane; receptor signaling protein activity; regulation of cell cycle |
| X56134 | [VIM](http://bioinfo.weizmann.ac.il/cards-bin/carddisp?VIM) | vimentin | cytoplasm; intermediate filament; protein binding; structural constituent of cytoskeleton |
| NM_002800 | [PSMB9](http://bioinfo.weizmann.ac.il/cards-bin/carddisp?PSMB9) | proteasome (prosome, macropain) subunit, beta type, 9 (large multifunctional protease 2) | endopeptidase activity; immune response; proteasome core complex (sensu Eukarya); proteolysis and peptidolysis; ubiquitin-dependent protein catabolism |
| J05593 | [TIMP2](http://bioinfo.weizmann.ac.il/cards-bin/carddisp?TIMP2) | tissue inhibitor of metalloproteinase 2 | extracellular matrix; metalloendopeptidase inhibitor activity |
| Y15227 | [DLEU1](http://bioinfo.weizmann.ac.il/cards-bin/carddisp?DLEU1) | deleted in lymphocytic leukemia, 1 | negative regulation of cell cycle |
| NC_004668.1 | EF0575 | cationic ABC transporter, ATP-binding protein | ATP binding; ATPase activity; nucleoside-triphosphatase activity; nucleotide binding |

Cluster S6

| ***GENBANK*** | ***SYMBOL*** | ***GENENAME*** | ***GENEONTOLOGY*** |
| --- | --- | --- | --- |
| M68520 | [CDK2](http://bioinfo.weizmann.ac.il/cards-bin/carddisp?CDK2) | cyclin-dependent kinase 2 | ATP binding; G2/M transition of mitotic cell cycle; cell cycle; cyclin-dependent protein kinase activity; cytokinesis; cytoplasm; mitosis; nucleus; positive regulation of cell proliferation; protein amino acid phosphorylation; protein serine/threonine kinase activity; regulation of DNA replication; transferase activity; traversing start control point of mitotic cell cycle |
| NM_016816 | [OAS1](http://bioinfo.weizmann.ac.il/cards-bin/carddisp?OAS1) | 2',5'-oligoadenylate synthetase 1, 40/46kDa | ATP binding; RNA binding; cytoplasm; immune response; nucleobase, nucleoside, nucleotide and nucleic acid metabolism; nucleotidyltransferase activity; response to virus; transferase activity |
| X05360 | [CDC2](http://bioinfo.weizmann.ac.il/cards-bin/carddisp?CDC2) | cell division cycle 2, G1 to S and G2 to M | ATP binding; cyclin-dependent protein kinase activity; cytokinesis; mitosis; nucleus; protein amino acid phosphorylation; transferase activity; traversing start control point of mitotic cell cycle |
| U82130 | [TSG101](http://bioinfo.weizmann.ac.il/cards-bin/carddisp?TSG101) | tumor susceptibility gene 101 | DNA binding; intracellular protein transport; protein transporter activity; regulation of cell growth; transcription corepressor activity; ubiquitin conjugating enzyme activity; ubiquitin cycle |
| AF000546 | [P2RY5](http://bioinfo.weizmann.ac.il/cards-bin/carddisp?P2RY5) | purinergic receptor P2Y, G-protein coupled, 5 | G-protein coupled receptor protein signaling pathway; integral to membrane; purinergic nucleotide receptor activity, G-protein coupled; rhodopsin-like receptor activity |
| D63878 | [NEDD5](http://bioinfo.weizmann.ac.il/cards-bin/carddisp?NEDD5) | neural precursor cell expressed, developmentally down-regulated 5 | GTP binding; GTPase activity; cell cycle; contractile ring; cytokinesis |
| Y07923 | [RND1](http://bioinfo.weizmann.ac.il/cards-bin/carddisp?RND1) | Rho family GTPase 1 | GTP binding; Rho small monomeric GTPase activity; actin filament organization; adherens junction; cytoskeleton; negative regulation of cell adhesion; neuronal remodeling; small GTPase mediated signal transduction |
| AF009510 | [TAPBP](http://bioinfo.weizmann.ac.il/cards-bin/carddisp?TAPBP) | TAP binding protein (tapasin) | Golgi membrane; MHC class I peptide loading complex; MHC class I protein binding; TAP1 binding; TAP2 binding; antigen processing, endogenous antigen via MHC class I; chaperone activity; endoplasmic reticulum membrane; immune response; integral to membrane; membrane; microsome; peptide antigen binding; peptide antigen stabilization; peptide antigen transporter activity; protein binding; protein complex assembly; retrograde transport, Golgi to ER |
| U03506 | [SLC1A1](http://bioinfo.weizmann.ac.il/cards-bin/carddisp?SLC1A1) | solute carrier family 1 (neuronal/epithelial high affinity glutamate transporter, system Xag), member 1 | L-glutamate transport; L-glutamate transporter activity; dicarboxylic acid transport; integral to plasma membrane; membrane fraction; sodium:dicarboxylate symporter activity; symporter activity; synaptic transmission; transport |
| X00497 | [CD74](http://bioinfo.weizmann.ac.il/cards-bin/carddisp?CD74) | CD74 antigen (invariant polypeptide of major histocompatibility complex, class II antigen-associated) | MHC class II receptor activity; chaperone activity; immune response; integral to membrane |
| AF010309 | [TP53I3](http://bioinfo.weizmann.ac.il/cards-bin/carddisp?TP53I3) | tumor protein p53 inducible protein 3 | alcohol dehydrogenase activity, zinc-dependent; biological_process unknown; cellular_component unknown; induction of apoptosis by oxidative stress; molecular_function unknown; zinc ion binding |
| M18082 | [SERPINB2](http://bioinfo.weizmann.ac.il/cards-bin/carddisp?SERPINB2) | serine (or cysteine) proteinase inhibitor, clade B (ovalbumin), member 2 | anti-apoptosis; plasminogen activator activity; serine-type endopeptidase inhibitor activity |
| NM_005564 | [LCN2](http://bioinfo.weizmann.ac.il/cards-bin/carddisp?LCN2) | lipocalin 2 (oncogene 24p3) | binding; cytoplasm; soluble fraction; transport; transporter activity |
| U90313 | [GSTO1](http://bioinfo.weizmann.ac.il/cards-bin/carddisp?GSTO1) | glutathione S-transferase omega 1 | biological_process unknown; cytoplasm; glutathione transferase activity; metabolism; monodehydroascorbate reductase (NADH) activity; transferase activity |
| U65410 | [MAD2L1](http://bioinfo.weizmann.ac.il/cards-bin/carddisp?MAD2L1) | MAD2 mitotic arrest deficient-like 1 (yeast) | cell cycle; kinetochore; mitosis; mitotic checkpoint; nucleus |
| U78095 | [SPINT2](http://bioinfo.weizmann.ac.il/cards-bin/carddisp?SPINT2) | serine protease inhibitor, Kunitz type, 2 | cell motility; extracellular; integral to membrane; serine-type endopeptidase inhibitor activity; soluble fraction |
| L07515 | [CBX5](http://bioinfo.weizmann.ac.il/cards-bin/carddisp?CBX5) | chromobox homolog 5 (HP1 alpha homolog, Drosophila) | chromatin; chromatin assembly/disassembly; chromatin binding; nuclear heterochromatin; nuclear membrane |
| NM_001907 | [CTRL](http://bioinfo.weizmann.ac.il/cards-bin/carddisp?CTRL) | chymotrypsin-like | chymotrypsin activity; digestion; extracellular space; hydrolase activity; peptidase activity; proteolysis and peptidolysis; trypsin activity |
| D83597 | [LY64](http://bioinfo.weizmann.ac.il/cards-bin/carddisp?LY64) | lymphocyte antigen 64 homolog, radioprotective 105kDa (mouse) | immune response; inflammatory response; integral to membrane; plasma membrane; protein binding; receptor activity |
| Y00503 | [KRT19](http://bioinfo.weizmann.ac.il/cards-bin/carddisp?KRT19) | keratin 19 | intermediate filament; structural constituent of cytoskeleton |
| M25269 | [ELK1](http://bioinfo.weizmann.ac.il/cards-bin/carddisp?ELK1) | ELK1, member of ETS oncogene family | nucleus; regulation of transcription, DNA-dependent; transcription factor activity |
| M21626 | [TRAV14DV4](http://bioinfo.weizmann.ac.il/cards-bin/carddisp?TRAV14DV4) | T cell receptor alpha variable 14/delta variable 4 |  |
| NM_003369 | [UVRAG](http://bioinfo.weizmann.ac.il/cards-bin/carddisp?UVRAG) | UV radiation resistance associated gene | Cytoplasm; DNA repair |
| D63878 | [DKFZp547A023](http://bioinfo.weizmann.ac.il/cards-bin/carddisp?DKFZp547A023) | hypothetical protein DKFZp547A023 |  |
| NM_000454 | [SOD1](http://bioinfo.weizmann.ac.il/cards-bin/carddisp?SOD1) | superoxide dismutase 1, soluble (amyotrophic lateral sclerosis 1 (adult)) | Copper; cytoplasm; superoxide metabolism; response to oxidative stress; neurogenesis |
