# Supplementary material for: Analysis of the interaction of extracellular matrix and phenotype of bladder cancer cells
Source: BMC Cancer. 2006 Jan 13;6:12. doi: 10.1186/1471-2407-6-12 (PMC1360102; doi:10.1186/1471-2407-6-12)
Supplement: Additional File 2 — Table 2. Hypervariable genes commonly expressed between different matrixes and their ontologies. GOTM results for this group of genes are shown at the bottom of the table. O:Observed gene number in the GO category; E:Expected gene number in the GO category; R:Ratio of enrichment for the GO category; P:Significance of enrichment for the GO category [file 1471-2407-6-12-S2.doc]

***Supplementary Table 2. Description of 20 Common Hypervariable Genes between Matrigel, Plastic and SISgel and their Gene Ontologies.***

| ***GENBANK*** | ***SYMBOL*** | ***GENENAME*** | ***GENEONTOLOGY*** |
| --- | --- | --- | --- |
| U13696 | [PMS2](http://bioinfo.weizmann.ac.il/cards-bin/carddisp?PMS2) | PMS2 postmeiotic segregation increased 2 (S. cerevisiae) | ATP binding; DNA binding; mismatch repair; negative regulation of cell cycle; nucleus |
| M68520 | [CDK2](http://bioinfo.weizmann.ac.il/cards-bin/carddisp?CDK2) | cyclin-dependent kinase 2 | ATP binding; G2/M transition of mitotic cell cycle; cell cycle; cyclin-dependent protein kinase activity; cytokinesis; cytoplasm; mitosis; nucleus; positive regulation of cell proliferation; protein amino acid phosphorylation; protein serine/threonine kinase activity; regulation of DNA replication; transferase activity; traversing start control point of mitotic cell cycle |
| X80692 | [MAPK6](http://bioinfo.weizmann.ac.il/cards-bin/carddisp?MAPK6) | mitogen-activated protein kinase 6 | ATP binding; MAP kinase activity; cell cycle; protein amino acid phosphorylation; protein serine/threonine kinase activity; signal transduction; transferase activity |
| X76104 | [DAPK1](http://bioinfo.weizmann.ac.il/cards-bin/carddisp?DAPK1) | death-associated protein kinase 1 | ATP binding; actin cytoskeleton; apoptosis; calcium/calmodulin-dependent protein kinase activity; calmodulin binding; calmodulin-dependent protein kinase I activity; induction of apoptosis by extracellular signals; protein amino acid phosphorylation; protein kinase cascade; protein serine/threonine kinase activity; signal transduction; transferase activity |
| S85655 | [PHB](http://bioinfo.weizmann.ac.il/cards-bin/carddisp?PHB) | prohibitin | DNA metabolism; cell growth and/or maintenance |
| AF000546 | [P2RY5](http://bioinfo.weizmann.ac.il/cards-bin/carddisp?P2RY5) | purinergic receptor P2Y, G-protein coupled, 5 | G-protein coupled receptor protein signaling pathway; integral to membrane; purinergic nucleotide receptor activity, G-protein coupled; rhodopsin-like receptor activity |
| M97934 | [STAT2](http://bioinfo.weizmann.ac.il/cards-bin/carddisp?STAT2) | signal transducer and activator of transcription 2, 113kDa | JAK-STAT cascade; hematopoietin/interferon-class (D200-domain) cytokine receptor signal transducer activity; intracellular signaling cascade; nucleus; regulation of transcription from Pol II promoter; signal transducer activity; transcription factor activity |
| X02851 | [IL1A](http://bioinfo.weizmann.ac.il/cards-bin/carddisp?IL1A) | interleukin 1, alpha | anti-apoptosis; apoptosis; cell proliferation; cell-cell signaling; chemotaxis; cytoplasm; extracellular space; immune response; inflammatory response; interleukin-1 receptor binding; negative regulation of cell proliferation; regulation of cell cycle; signal transducer activity |
| X86779 | [FASTK](http://bioinfo.weizmann.ac.il/cards-bin/carddisp?FASTK) | FAST kinase | apoptosis; induction of apoptosis by extracellular signals; kinase activity; protein amino acid phosphorylation; protein serine/threonine kinase activity; signal transduction; transferase activity |
| U56390 | [CASP9](http://bioinfo.weizmann.ac.il/cards-bin/carddisp?CASP9) | caspase 9, apoptosis-related cysteine protease | apoptotic program; caspase activation via cytochrome c; caspase activity; enzyme activator activity; intracellular; protein binding; proteolysis and peptidolysis; regulation of apoptosis |
| U60520 | [CASP8](http://bioinfo.weizmann.ac.il/cards-bin/carddisp?CASP8) | caspase 8, apoptosis-related cysteine protease | apoptotic program; caspase activity; cysteine-type peptidase activity; cytoskeleton; hydrolase activity; mitochondrion; protein binding; proteolysis and peptidolysis; regulation of apoptosis |
| X57766 | [MMP11](http://bioinfo.weizmann.ac.il/cards-bin/carddisp?MMP11) | matrix metalloproteinase 11 (stromelysin 3) | calcium ion binding; collagen catabolism; extracellular matrix; hydrolase activity; morphogenesis; stromelysin 3 activity; zinc ion binding |
| M55172 | [AGC1](http://bioinfo.weizmann.ac.il/cards-bin/carddisp?AGC1) | aggrecan 1 (chondroitin sulfate proteoglycan 1, large aggregating proteoglycan, antigen identified by monoclonal antibody A0122) | cell adhesion; extracellular matrix; heterophilic cell adhesion; hyaluronic acid binding; sugar binding |
| X03168 | [VTN](http://bioinfo.weizmann.ac.il/cards-bin/carddisp?VTN) | vitronectin (serum spreading factor, somatomedin B, complement S-protein) | cell adhesion; extracellular space; heparin binding; immune response; protein binding |
| U65410 | [MAD2L1](http://bioinfo.weizmann.ac.il/cards-bin/carddisp?MAD2L1) | MAD2 mitotic arrest deficient-like 1 (yeast) | cell cycle; kinetochore; mitosis; mitotic checkpoint; nucleus |
| X06374 | [PDGFA](http://bioinfo.weizmann.ac.il/cards-bin/carddisp?PDGFA) | platelet-derived growth factor alpha polypeptide | cell proliferation; cell surface receptor linked signal transduction; cell-cell signaling; extracellular space; growth factor activity; membrane; platelet-derived growth factor receptor binding; regulation of cell cycle |
| L27943 | [CDA](http://bioinfo.weizmann.ac.il/cards-bin/carddisp?CDA) | cytidine deaminase | cytidine deaminase activity; cytidine metabolism; hydrolase activity; nucleobase, nucleoside, nucleotide and nucleic acid metabolism; zinc ion binding |
| X03124 | [TIMP1](http://bioinfo.weizmann.ac.il/cards-bin/carddisp?TIMP1) | tissue inhibitor of metalloproteinase 1 (erythroid potentiating activity, collagenase inhibitor) | development; extracellular matrix; metalloendopeptidase inhibitor activity; metallopeptidase activity; positive regulation of cell proliferation; proteolysis and peptidolysis |
| A14844 | [IL2](http://www.ncbi.nlm.nih.gov/entrez/query.fcgi?db=gene&cmd=Retrieve&dopt=Graphics&list_uids=3558) | interleukin 2 | cytokine activity; interleukin-2 receptor binding; extracellular space; anti-apoptosis; immune response; cell adhesion; cell-cell signaling; positive regulation of cell proliferation; kinase activator activity; antimicrobial humoral response (sensu Vertebrata); natural killer cell activation; T-cell differentiation; positive regulation of cell growth |
| M26326 | [KRT18](http://www.ncbi.nlm.nih.gov/entrez/query.fcgi?db=gene&cmd=Retrieve&dopt=Graphics&list_uids=3875) | keratin 18 | structural molecule activity; structural constituent of cytoskeleton; intermediate filament; morphogenesis |

GOTM

[protein kinase cascade*(O=3;E=0.42;R=7.14;P=0.00803918357424)](http://genereg.ornl.gov/gotm/node_id_list_new.php?gotree_id=01-03-01-06-02-11)

[1612](http://genereg.ornl.gov/gotm/llid_info.php?llid=1612)(DAPK1)
[6773](http://genereg.ornl.gov/gotm/llid_info.php?llid=6773)(STAT2)
[841](http://genereg.ornl.gov/gotm/llid_info.php?llid=841)(CASP8)

[regulation of cell cycle*(O=6;E=0.67;R=8.96;P=3.49879988628E-05)](http://genereg.ornl.gov/gotm/node_id_list_new.php?gotree_id=01-03-03-03-09)

[1017](http://genereg.ornl.gov/gotm/llid_info.php?llid=1017)(CDK2)
[3552](http://genereg.ornl.gov/gotm/llid_info.php?llid=3552)(IL1A)
[4085](http://genereg.ornl.gov/gotm/llid_info.php?llid=4085)(MAD2L1)
[5154](http://genereg.ornl.gov/gotm/llid_info.php?llid=5154)(PDGFA)
[5245](http://genereg.ornl.gov/gotm/llid_info.php?llid=5245)(PHB)
[5395](http://genereg.ornl.gov/gotm/llid_info.php?llid=5395)(PMS2)

[apoptotic program*(O=2;E=0.08;R=25;P=0.00252652679595)](http://genereg.ornl.gov/gotm/node_id_list_new.php?gotree_id=01-03-03-04-04-01-02)

[841](http://genereg.ornl.gov/gotm/llid_info.php?llid=841)(CASP8)
[842](http://genereg.ornl.gov/gotm/llid_info.php?llid=842)(CASP9)

[induction of apoptosis by extracellular signals*(O=2;E=0.05;R=40;P=0.00105828385563)](http://genereg.ornl.gov/gotm/node_id_list_new.php?gotree_id=01-03-03-04-04-01-11-02-01-02)

[10922](http://genereg.ornl.gov/gotm/llid_info.php?llid=10922)(FASTK)
[1612](http://genereg.ornl.gov/gotm/llid_info.php?llid=1612)(DAPK1)

[positive regulation of cell proliferation*(O=3;E=0.19;R=15.79;P=0.000825175755169)](http://genereg.ornl.gov/gotm/node_id_list_new.php?gotree_id=01-03-03-10-11-02)

[1017](http://genereg.ornl.gov/gotm/llid_info.php?llid=1017)(CDK2)
[3558](http://genereg.ornl.gov/gotm/llid_info.php?llid=3558)(IL2)
[7076](http://genereg.ornl.gov/gotm/llid_info.php?llid=7076)(TIMP1)

[negative regulation of cellular physiological process*(O=5;E=0.82;R=6.1;P=0.00105915545688)](http://genereg.ornl.gov/gotm/node_id_list_new.php?gotree_id=01-03-03-21-01)

[3552](http://genereg.ornl.gov/gotm/llid_info.php?llid=3552)(IL1A)
[3558](http://genereg.ornl.gov/gotm/llid_info.php?llid=3558)(IL2)
[5245](http://genereg.ornl.gov/gotm/llid_info.php?llid=5245)(PHB)
[5395](http://genereg.ornl.gov/gotm/llid_info.php?llid=5395)(PMS2)
[7076](http://genereg.ornl.gov/gotm/llid_info.php?llid=7076)(TIMP1)

[polysaccharide binding*(O=2;E=0.12;R=16.67;P=0.00625162260473)](http://genereg.ornl.gov/gotm/node_id_list_new.php?gotree_id=02-02-28-04)

[176](http://genereg.ornl.gov/gotm/llid_info.php?llid=176)(AGC1)
[7448](http://genereg.ornl.gov/gotm/llid_info.php?llid=7448)(VTN)

[extracellular space*(O=4;E=0.67;R=5.97;P=0.00361838102414)](http://genereg.ornl.gov/gotm/node_id_list_new.php?gotree_id=03-04-04)

[3552](http://genereg.ornl.gov/gotm/llid_info.php?llid=3552)(IL1A)
[3558](http://genereg.ornl.gov/gotm/llid_info.php?llid=3558)(IL2)
[5154](http://genereg.ornl.gov/gotm/llid_info.php?llid=5154)(PDGFA)
[7448](http://genereg.ornl.gov/gotm/llid_info.php?llid=7448)(VTN)
