# Supplementary material for: Analysis of the interaction of extracellular matrix and phenotype of bladder cancer cells
Source: BMC Cancer. 2006 Jan 13;6:12. doi: 10.1186/1471-2407-6-12 (PMC1360102; doi:10.1186/1471-2407-6-12)
Supplement: Additional File 3 — Table 3. Hypervariable genes commonly expressed between SISgel and Matrigel and their ontologies. GOTM results for this group of genes are shown at the bottom of the table. O:Observed gene number in the GO category; E:Expected gene number in the GO category; R:Ratio of enrichment for the GO category; P:Significance of enrichment for the GO category [file 1471-2407-6-12-S3.doc]

***Supplementary Table 3. Description of 33 Common Hypervariable Genes between Matrigel and SISgel and their Gene Ontologies.***

| ***GENBANK*** | ***SYMBOL*** | ***GENENAME*** | ***GENEONTOLOGY*** |
| --- | --- | --- | --- |
| U13695 | [PMS1](http://bioinfo.weizmann.ac.il/cards-bin/carddisp?PMS1) | PMS1 postmeiotic segregation increased 1 (S. cerevisiae) | ATP binding; DNA binding; mismatch repair; negative regulation of cell cycle; nucleus; regulation of transcription, DNA-dependent |
| M63167 | [AKT1](http://bioinfo.weizmann.ac.il/cards-bin/carddisp?AKT1) | v-akt murine thymoma viral oncogene homolog 1 | ATP binding; G-protein coupled receptor protein signaling pathway; anti-apoptosis; nitric oxide biosynthesis; nucleus; protein amino acid phosphorylation; receptor signaling protein serine/threonine kinase activity; response to heat; signal transduction; transferase activity |
| M11810 | [OAS1](http://bioinfo.weizmann.ac.il/cards-bin/carddisp?OAS1) | 2',5'-oligoadenylate synthetase 1, 40/46kDa | ATP binding; RNA binding; cytoplasm; immune response; nucleobase, nucleoside, nucleotide and nucleic acid metabolism; nucleotidyltransferase activity; response to virus; transferase activity |
| X05360 | [CDC2](http://bioinfo.weizmann.ac.il/cards-bin/carddisp?CDC2) | cell division cycle 2, G1 to S and G2 to M | ATP binding; cyclin-dependent protein kinase activity; cytokinesis; mitosis; nucleus; protein amino acid phosphorylation; transferase activity; traversing start control point of mitotic cell cycle |
| M32865 | [G22P1](http://bioinfo.weizmann.ac.il/cards-bin/carddisp?G22P1) | thyroid autoantigen 70kDa (Ku antigen) | ATP-dependent DNA helicase activity; DNA ligation; double-strand break repair via nonhomologous end-joining; double-stranded DNA binding; helicase activity; membrane fraction; nucleus |
| M57627 | [IL10](http://bioinfo.weizmann.ac.il/cards-bin/carddisp?IL10) | interleukin 10 | B-cell differentiation; B-cell proliferation; T-helper 2 type immune response; anti-apoptosis; cell-cell signaling; cytokine activity; cytoplasmic sequestering of NF-kappaB; extracellular; hemopoiesis; immune cell chemotaxis; immune response; interleukin-10 receptor binding; negative regulation of MHC class II biosynthesis; negative regulation of T-cell proliferation; negative regulation of interferon-alpha biosynthesis; negative regulation of interferon-gamma biosynthesis; negative regulation of nitric oxide biosynthesis; regulation of isotype switching |
| S74678 | [HNRPK](http://bioinfo.weizmann.ac.il/cards-bin/carddisp?HNRPK) | heterogeneous nuclear ribonucleoprotein K | DNA binding; RNA binding; RNA processing; heterogeneous nuclear ribonucleoprotein complex; nucleoplasm |
| U69127 | [FUBP3](http://bioinfo.weizmann.ac.il/cards-bin/carddisp?FUBP3) | far upstream element (FUSE) binding protein 3 | DNA binding; nucleus; regulation of transcription, DNA-dependent |
| D17516 | [ADCYAP1R1](http://bioinfo.weizmann.ac.il/cards-bin/carddisp?ADCYAP1R1) | adenylate cyclase activating polypeptide 1 (pituitary) receptor type I | G-protein coupled receptor activity; G-protein coupled receptor protein signaling pathway; integral to plasma membrane; receptor activity; spermatogenesis; vasoactive intestinal polypeptide receptor activity |
| U22398 | [CDKN1C](http://bioinfo.weizmann.ac.il/cards-bin/carddisp?CDKN1C) | cyclin-dependent kinase inhibitor 1C (p57, Kip2) | G1 phase of mitotic cell cycle; cell cycle; cell cycle arrest; cyclin-dependent protein kinase inhibitor activity; negative regulation of cell cycle; negative regulation of cell proliferation; nucleus; regulation of CDK activity |
| L25081 | [ARHC](http://bioinfo.weizmann.ac.il/cards-bin/carddisp?ARHC) | ras homolog gene family, member C | GTP binding; Rho small monomeric GTPase activity; catalytic activity; cell growth and/or maintenance; small GTPase mediated signal transduction |
| M35416 | [RALB](http://bioinfo.weizmann.ac.il/cards-bin/carddisp?RALB) | v-ral simian leukemia viral oncogene homolog B (ras related; GTP binding protein) | GTP binding; signal transduction; small GTPase mediated signal transduction; small monomeric GTPase activity |
| M11886 | [HLA-C](http://bioinfo.weizmann.ac.il/cards-bin/carddisp?HLA-C) | major histocompatibility complex, class I, C | MHC class I receptor activity; MHC class II receptor activity; antigen presentation, endogenous antigen; antigen processing, endogenous antigen via MHC class I; immune response; integral to membrane |
| X74295 | [MGC17301](http://bioinfo.weizmann.ac.il/cards-bin/carddisp?MGC17301) | hypothetical protein MGC17301 | S-adenosylmethionine-dependent methyltransferase activity |
| X02812 | [TGFB1](http://bioinfo.weizmann.ac.il/cards-bin/carddisp?TGFB1) | transforming growth factor, beta 1 (Camurati-Engelmann disease) | anti-apoptosis; cell growth; cell proliferation; cell-cell signaling; growth; regulation of cell cycle; transforming growth factor beta receptor binding; transforming growth factor beta receptor signaling pathway |
| M18082 | [SERPINB2](http://bioinfo.weizmann.ac.il/cards-bin/carddisp?SERPINB2) | serine (or cysteine) proteinase inhibitor, clade B (ovalbumin), member 2 | anti-apoptosis; plasminogen activator activity; serine-type endopeptidase inhibitor activity |
| K02770 | [IL1B](http://bioinfo.weizmann.ac.il/cards-bin/carddisp?IL1B) | interleukin 1, beta | antimicrobial humoral response (sensu Vertebrata); apoptosis; cell proliferation; cell-cell signaling; extracellular space; immune response; inflammatory response; interleukin-1 receptor binding; negative regulation of cell proliferation; regulation of cell cycle; signal transducer activity; signal transduction |
| U60519 | [CASP10](http://bioinfo.weizmann.ac.il/cards-bin/carddisp?CASP10) | caspase 10, apoptosis-related cysteine protease | caspase activity; cysteine-type peptidase activity; hydrolase activity; induction of apoptosis; peptidase activity; protein binding; proteolysis and peptidolysis; regulation of apoptosis |
| D13866 | [CTNNA1](http://bioinfo.weizmann.ac.il/cards-bin/carddisp?CTNNA1) | catenin (cadherin-associated protein), alpha 1, 102kDa | cell adhesion; cytoskeleton; protein binding; structural molecule activity |
| U09579 | [CDKN1A](http://bioinfo.weizmann.ac.il/cards-bin/carddisp?CDKN1A) | cyclin-dependent kinase inhibitor 1A (p21, Cip1) | cell cycle arrest; cyclin-dependent protein kinase inhibitor activity; induction of apoptosis by intracellular signals; kinase activity; negative regulation of cell proliferation; nucleus; protein kinase activity; regulation of CDK activity |
| U78095 | [SPINT2](http://bioinfo.weizmann.ac.il/cards-bin/carddisp?SPINT2) | serine protease inhibitor, Kunitz type, 2 | cell motility; extracellular; integral to membrane; serine-type endopeptidase inhibitor activity; soluble fraction |
| U53446 | [DAB2](http://bioinfo.weizmann.ac.il/cards-bin/carddisp?DAB2) | disabled homolog 2, mitogen-responsive phosphoprotein (Drosophila) | cell proliferation |
| X02811 | [PDGFB](http://bioinfo.weizmann.ac.il/cards-bin/carddisp?PDGFB) | platelet-derived growth factor beta polypeptide (simian sarcoma viral (v-sis) oncogene homolog) | cell proliferation; extracellular; growth factor activity; membrane; platelet-derived growth factor receptor binding; regulation of cell cycle; response to wounding |
| X91940 | [WNT8B](http://bioinfo.weizmann.ac.il/cards-bin/carddisp?WNT8B) | wingless-type MMTV integration site family, member 8B | cell-cell signaling; development; extracellular; frizzled-2 signaling pathway; neurogenesis; signal transducer activity; signal transduction |
| X74295 | [ITGA7](http://bioinfo.weizmann.ac.il/cards-bin/carddisp?ITGA7) | integrin, alpha 7 | cell-matrix adhesion; cellular morphogenesis; homophilic cell adhesion; integrin complex; integrin-mediated signaling pathway; muscle development; protein binding; receptor activity |
| M30938 | [XRCC5](http://bioinfo.weizmann.ac.il/cards-bin/carddisp?XRCC5) | X-ray repair complementing defective repair in Chinese hamster cells 5 (double-strand-break rejoining; Ku autoantigen, 80kDa) | cellular_component unknown; molecular_function unknown; regulation of DNA repair |
| L07515 | [CBX5](http://bioinfo.weizmann.ac.il/cards-bin/carddisp?CBX5) | chromobox homolog 5 (HP1 alpha homolog, Drosophila) | chromatin; chromatin assembly/disassembly; chromatin binding; nuclear heterochromatin; nuclear membrane |
| M34225 | [KRT8](http://bioinfo.weizmann.ac.il/cards-bin/carddisp?KRT8) | keratin 8 | cytoskeleton organization and biogenesis; intermediate filament; phosphorylation; structural molecule activity |
| J05593 | [TIMP2](http://bioinfo.weizmann.ac.il/cards-bin/carddisp?TIMP2) | tissue inhibitor of metalloproteinase 2 | extracellular matrix; metalloendopeptidase inhibitor activity |
| D83597 | [LY64](http://bioinfo.weizmann.ac.il/cards-bin/carddisp?LY64) | lymphocyte antigen 64 homolog, radioprotective 105kDa (mouse) | immune response; inflammatory response; integral to membrane; plasma membrane; protein binding; receptor activity |
| M82882 | [ELF1](http://bioinfo.weizmann.ac.il/cards-bin/carddisp?ELF1) | E74-like factor 1 (ets domain transcription factor) | nucleus; regulation of transcription, DNA-dependent; transcription factor activity; transcriptional activator activity |
| J00209 | [IFNA10](http://www.ncbi.nlm.nih.gov/entrez/query.fcgi?db=gene&cmd=Retrieve&dopt=Graphics&list_uids=3446) | interferon, alpha 10 | interferon-alpha/beta receptor binding; extracellular region; defense response; response to virus |
| U09825 | [TRIM26](http://www.ncbi.nlm.nih.gov/entrez/query.fcgi?db=gene&cmd=Retrieve&dopt=Graphics&list_uids=7726) | tripartite motif-containing 26 | ubiquitin ligase complex; DNA binding; ubiquitin-protein ligase activity; protein binding; zinc ion binding; protein ubiquitination; metal ion binding |

GOTM

[negative regulation of cell proliferation*(O=4;E=0.27;R=14.81;P=0.000143357846318)](http://genereg.ornl.gov/gotm/node_id_list_new.php?gotree_id=01-08-01-03-03-08)

[1026](http://genereg.ornl.gov/gotm/llid_info.php?llid=1026)(CDKN1A)
[1028](http://genereg.ornl.gov/gotm/llid_info.php?llid=1028)(CDKN1C)
[3553](http://genereg.ornl.gov/gotm/llid_info.php?llid=3553)(IL1B)
[3586](http://genereg.ornl.gov/gotm/llid_info.php?llid=3586)(IL10)

[anti-apoptosis*(O=4;E=0.19;R=21.05;P=3.82042266694E-05)](http://genereg.ornl.gov/gotm/node_id_list_new.php?gotree_id=01-08-01-03-03-17-01-01)

[1026](http://genereg.ornl.gov/gotm/llid_info.php?llid=1026)(CDKN1A)
[1028](http://genereg.ornl.gov/gotm/llid_info.php?llid=1028)(CDKN1C)
[3553](http://genereg.ornl.gov/gotm/llid_info.php?llid=3553)(IL1B)
[3586](http://genereg.ornl.gov/gotm/llid_info.php?llid=3586)(IL10)

[cyclin-dependent protein kinase inhibitor activity*(O=2;E=0.02;R=100;P=9.46812599264E-05)](http://genereg.ornl.gov/gotm/node_id_list_new.php?gotree_id=02-05-04-10-01-03)

[1026](http://genereg.ornl.gov/gotm/llid_info.php?llid=1026)(CDKN1A)
[1028](http://genereg.ornl.gov/gotm/llid_info.php?llid=1028)(CDKN1C)

[endopeptidase inhibitor activity*(O=3;E=0.26;R=11.54;P=0.00209820306256)](http://genereg.ornl.gov/gotm/node_id_list_new.php?gotree_id=02-05-04-16-03)

[10653](http://genereg.ornl.gov/gotm/llid_info.php?llid=10653)(SPINT2)
[5055](http://genereg.ornl.gov/gotm/llid_info.php?llid=5055)(SERPINB2)
[7077](http://genereg.ornl.gov/gotm/llid_info.php?llid=7077)(TIMP2)

[extracellular region*(O=7;E=2.28;R=3.07;P=0.00545647257768)](http://genereg.ornl.gov/gotm/node_id_list_new.php?gotree_id=03-04)

[10653](http://genereg.ornl.gov/gotm/llid_info.php?llid=10653)(SPINT2)
[3553](http://genereg.ornl.gov/gotm/llid_info.php?llid=3553)(IL1B)
[3586](http://genereg.ornl.gov/gotm/llid_info.php?llid=3586)(IL10)
[5055](http://genereg.ornl.gov/gotm/llid_info.php?llid=5055)(SERPINB2)
[5155](http://genereg.ornl.gov/gotm/llid_info.php?llid=5155)(PDGFB)
[7077](http://genereg.ornl.gov/gotm/llid_info.php?llid=7077)(TIMP2)
[7479](http://genereg.ornl.gov/gotm/llid_info.php?llid=7479)(WNT8B)
