# Supplementary material for: Analysis of the interaction of extracellular matrix and phenotype of bladder cancer cells
Source: BMC Cancer. 2006 Jan 13;6:12. doi: 10.1186/1471-2407-6-12 (PMC1360102; doi:10.1186/1471-2407-6-12)
Supplement: Additional File 4 — Table 4. Genes driving significant ontologies on clusters on different matrixes. O:Observed gene number in the GO category; E:Expected gene number in the GO category; R:Ratio of enrichment for the GO category; P:Significance of enrichment for the GO category [file 1471-2407-6-12-S4.doc]

***Supplementary Table 4. Gene Ontologies of Individual Clusters.***

| ***Cluster*** | ***Ontology and gene symbols*** |
| --- | --- |
| Matrigel_1 | [negative regulation of biological process*(O=4;E=0.55;R=7.27;P=0.00155586293923)](http://genereg.ornl.gov/gotm/node_id_list_new.php?gotree_id=01-08-01)  [396](http://genereg.ornl.gov/gotm/llid_info.php?llid=396)(ARHGDIA) [5055](http://genereg.ornl.gov/gotm/llid_info.php?llid=5055)(SERPINB2) [5062](http://genereg.ornl.gov/gotm/llid_info.php?llid=5062)(PAK2) [5328](http://genereg.ornl.gov/gotm/llid_info.php?llid=5328)(PLAU) |
| Matrigel_2 | [mRNA processing*(O=3;E=0.3;R=10;P=0.00304606084754)](http://genereg.ornl.gov/gotm/node_id_list_new.php?gotree_id=01-03-03-11-23-13-02-04)  [2926](http://genereg.ornl.gov/gotm/llid_info.php?llid=2926)(GRSF1) [4686](http://genereg.ornl.gov/gotm/llid_info.php?llid=4686)(NCBP1) [9984](http://genereg.ornl.gov/gotm/llid_info.php?llid=9984)(THOC1) |
| Matrigel_3 | [mismatch repair*(O=2;E=0.02;R=100;P=0.000199441323379)](http://genereg.ornl.gov/gotm/node_id_list_new.php?gotree_id=01-03-03-11-23-01-09-03-07-02)  [5378](http://genereg.ornl.gov/gotm/llid_info.php?llid=5378)(PMS1) [5395](http://genereg.ornl.gov/gotm/llid_info.php?llid=5395)(PMS2  [transcription factor activity*(O=6;E=0.91;R=6.59;P=0.000142427094418)](http://genereg.ornl.gov/gotm/node_id_list_new.php?gotree_id=02-02-22-01-21)  [1958](http://genereg.ornl.gov/gotm/llid_info.php?llid=1958)(EGR1) [1997](http://genereg.ornl.gov/gotm/llid_info.php?llid=1997)(ELF1) [3065](http://genereg.ornl.gov/gotm/llid_info.php?llid=3065)(HDAC1) [5813](http://genereg.ornl.gov/gotm/llid_info.php?llid=5813)(PURA) [6773](http://genereg.ornl.gov/gotm/llid_info.php?llid=6773)(STAT2) [8939](http://genereg.ornl.gov/gotm/llid_info.php?llid=8939)(FUBP3) |
| Matrigel_4 | [anti-apoptosis*(O=3;E=0.19;R=15.79;P=0.000923178895937)](http://genereg.ornl.gov/gotm/node_id_list_new.php?gotree_id=01-03-03-04-04-01-11-01-01)  [207](http://genereg.ornl.gov/gotm/llid_info.php?llid=207)(AKT1) [573](http://genereg.ornl.gov/gotm/llid_info.php?llid=573)(BAG1) [7040](http://genereg.ornl.gov/gotm/llid_info.php?llid=7040)(TGFB1)  [induction of apoptosis by extracellular signals*(O=3;E=0.06;R=50;P=2.88896483242E-05)](http://genereg.ornl.gov/gotm/node_id_list_new.php?gotree_id=01-03-03-04-04-01-11-02-01-02)  [10922](http://genereg.ornl.gov/gotm/llid_info.php?llid=10922)(FASTK) [1612](http://genereg.ornl.gov/gotm/llid_info.php?llid=1612)(DAPK1) [8738](http://genereg.ornl.gov/gotm/llid_info.php?llid=8738)(CRADD)  [protein binding*(O=12;E=6;R=2;P=0.00844711907163)](http://genereg.ornl.gov/gotm/node_id_list_new.php?gotree_id=02-02-35)  [10987](http://genereg.ornl.gov/gotm/llid_info.php?llid=10987)(COPS5) [1434](http://genereg.ornl.gov/gotm/llid_info.php?llid=1434)(CSE1L) [1612](http://genereg.ornl.gov/gotm/llid_info.php?llid=1612)(DAPK1) [2335](http://genereg.ornl.gov/gotm/llid_info.php?llid=2335)(FN1) [573](http://genereg.ornl.gov/gotm/llid_info.php?llid=573)(BAG1) [7040](http://genereg.ornl.gov/gotm/llid_info.php?llid=7040)(TGFB1) [7133](http://genereg.ornl.gov/gotm/llid_info.php?llid=7133)(TNFRSF1B) [7158](http://genereg.ornl.gov/gotm/llid_info.php?llid=7158)(TP53BP1) [7189](http://genereg.ornl.gov/gotm/llid_info.php?llid=7189)(TRAF6) [7726](http://genereg.ornl.gov/gotm/llid_info.php?llid=7726)(TRIM26) [843](http://genereg.ornl.gov/gotm/llid_info.php?llid=843)(CASP10) [8738](http://genereg.ornl.gov/gotm/llid_info.php?llid=8738)(CRADD)  [protein serine/threonine kinase activity*(O=5;E=0.86;R=5.81;P=0.00144720065504)](http://genereg.ornl.gov/gotm/node_id_list_new.php?gotree_id=02-03-40-16-05-77-05)  [10922](http://genereg.ornl.gov/gotm/llid_info.php?llid=10922)(FASTK) [1196](http://genereg.ornl.gov/gotm/llid_info.php?llid=1196)(CLK2) [1612](http://genereg.ornl.gov/gotm/llid_info.php?llid=1612)(DAPK1) [207](http://genereg.ornl.gov/gotm/llid_info.php?llid=207)(AKT1) [5610](http://genereg.ornl.gov/gotm/llid_info.php?llid=5610)(EIF2AK2) |
| Matrigel_5 | [regulation of cyclin dependent protein kinase activity*(O=2;E=0.06;R=33.33;P=0.00167380108306)](http://genereg.ornl.gov/gotm/node_id_list_new.php?gotree_id=01-03-03-03-05-05-02-02)  [1028](http://genereg.ornl.gov/gotm/llid_info.php?llid=1028)(CDKN1C) [900](http://genereg.ornl.gov/gotm/llid_info.php?llid=900)(CCNG1)  [mitotic control*(O=4;E=0.22;R=18.18;P=5.56893362383E-05)](http://genereg.ornl.gov/gotm/node_id_list_new.php?gotree_id=01-03-03-03-06-03-03)  [1017](http://genereg.ornl.gov/gotm/llid_info.php?llid=1017)(CDK2) [4085](http://genereg.ornl.gov/gotm/llid_info.php?llid=4085)(MAD2L1) [900](http://genereg.ornl.gov/gotm/llid_info.php?llid=900)(CCNG1) [983](http://genereg.ornl.gov/gotm/llid_info.php?llid=983)(CDC2)  [negative regulation of cell proliferation*(O=4;E=0.22;R=18.18;P=5.72805570642E-05)](http://genereg.ornl.gov/gotm/node_id_list_new.php?gotree_id=01-03-03-21-01-08)  [1028](http://genereg.ornl.gov/gotm/llid_info.php?llid=1028)(CDKN1C) [10668](http://genereg.ornl.gov/gotm/llid_info.php?llid=10668)(CGRRF1) [4831](http://genereg.ornl.gov/gotm/llid_info.php?llid=4831)(NME2) [5245](http://genereg.ornl.gov/gotm/llid_info.php?llid=5245)(PHB) |
| Matrigel_6 |  |
| Matrigel_7 | [regulation of cyclin dependent protein kinase activity*(O=2;E=0.06;R=33.33;P=0.00150931816125)](http://genereg.ornl.gov/gotm/node_id_list_new.php?gotree_id=01-03-03-03-05-05-02-02)  [1026](http://genereg.ornl.gov/gotm/llid_info.php?llid=1026)(CDKN1A) [993](http://genereg.ornl.gov/gotm/llid_info.php?llid=993)(CDC25A)  [anti-apoptosis*(O=3;E=0.15;R=20;P=0.000402304954405)](http://genereg.ornl.gov/gotm/node_id_list_new.php?gotree_id=01-03-03-04-04-01-11-01-01)  [3552](http://genereg.ornl.gov/gotm/llid_info.php?llid=3552)(IL1A) [3558](http://genereg.ornl.gov/gotm/llid_info.php?llid=3558)(IL2) [3586](http://genereg.ornl.gov/gotm/llid_info.php?llid=3586)(IL10)  [negative regulation of cell proliferation*(O=4;E=0.21;R=19.05;P=4.62152897052E-05)](http://genereg.ornl.gov/gotm/node_id_list_new.php?gotree_id=01-03-03-10-11-01)  [1026](http://genereg.ornl.gov/gotm/llid_info.php?llid=1026)(CDKN1A) [3552](http://genereg.ornl.gov/gotm/llid_info.php?llid=3552)(IL1A) [3553](http://genereg.ornl.gov/gotm/llid_info.php?llid=3553)(IL1B) [3586](http://genereg.ornl.gov/gotm/llid_info.php?llid=3586)(IL10)  [positive regulation of cell proliferation*(O=3;E=0.18;R=16.67;P=0.000706244892571)](http://genereg.ornl.gov/gotm/node_id_list_new.php?gotree_id=01-03-03-10-11-02)  [3558](http://genereg.ornl.gov/gotm/llid_info.php?llid=3558)(IL2) [7076](http://genereg.ornl.gov/gotm/llid_info.php?llid=7076)(TIMP1) [994](http://genereg.ornl.gov/gotm/llid_info.php?llid=994)(CDC25B)  [extracellular space*(O=7;E=0.71;R=9.86;P=2.80359713907E-06)](http://genereg.ornl.gov/gotm/node_id_list_new.php?gotree_id=03-04-04)  [3440](http://genereg.ornl.gov/gotm/llid_info.php?llid=3440)(IFNA2) [3552](http://genereg.ornl.gov/gotm/llid_info.php?llid=3552)(IL1A) [3553](http://genereg.ornl.gov/gotm/llid_info.php?llid=3553)(IL1B) [3558](http://genereg.ornl.gov/gotm/llid_info.php?llid=3558)(IL2) [3586](http://genereg.ornl.gov/gotm/llid_info.php?llid=3586)(IL10) [5154](http://genereg.ornl.gov/gotm/llid_info.php?llid=5154)(PDGFA) [7448](http://genereg.ornl.gov/gotm/llid_info.php?llid=7448)(VTN) |
| Plastic_1 | [induction of apoptosis*(O=2;E=0.07;R=28.57;P=0.00209604041352)](http://genereg.ornl.gov/gotm/node_id_list_new.php?gotree_id=01-03-03-21-20-02-02-01)  [5625](http://genereg.ornl.gov/gotm/llid_info.php?llid=5625)(PRODH) [837](http://genereg.ornl.gov/gotm/llid_info.php?llid=837)(CASP4) |
| Plastic_2 | [immune response*(O=3;E=0.3;R=10;P=0.00203304045492)](http://genereg.ornl.gov/gotm/node_id_list_new.php?gotree_id=01-07-15-01-02-07)  [3434](http://genereg.ornl.gov/gotm/llid_info.php?llid=3434)(IFIT1) [397](http://genereg.ornl.gov/gotm/llid_info.php?llid=397)(ARHGDIB) [4258](http://genereg.ornl.gov/gotm/llid_info.php?llid=4258)(MGST2) |
| Plastic_3 | [negative regulation of cell cycle*(O=2;E=0.11;R=18.18;P=0.00554709510197)](http://genereg.ornl.gov/gotm/node_id_list_new.php?gotree_id=01-03-03-03-09-04)  [10301](http://genereg.ornl.gov/gotm/llid_info.php?llid=10301)(DLEU1) [4292](http://genereg.ornl.gov/gotm/llid_info.php?llid=4292)(MLH1) |
| Plastic_4 | [protein kinase cascade*(O=4;E=0.63;R=6.35;P=0.00340433011996)](http://genereg.ornl.gov/gotm/node_id_list_new.php?gotree_id=01-03-01-06-02-11)  [1612](http://genereg.ornl.gov/gotm/llid_info.php?llid=1612)(DAPK1) [1616](http://genereg.ornl.gov/gotm/llid_info.php?llid=1616)(DAXX) [6773](http://genereg.ornl.gov/gotm/llid_info.php?llid=6773)(STAT2) [841](http://genereg.ornl.gov/gotm/llid_info.php?llid=841)(CASP8)  [meiotic recombination*(O=2;E=0.04;R=50;P=0.000893147040106)](http://genereg.ornl.gov/gotm/node_id_list_new.php?gotree_id=01-03-03-03-06-02-03-05-12)  [472](http://genereg.ornl.gov/gotm/llid_info.php?llid=472)(ATM) [5885](http://genereg.ornl.gov/gotm/llid_info.php?llid=5885)(RAD21)  [cell cycle arrest*(O=2;E=0.15;R=13.33;P=0.00956671174217)](http://genereg.ornl.gov/gotm/node_id_list_new.php?gotree_id=01-03-03-03-09-01)  [5608](http://genereg.ornl.gov/gotm/llid_info.php?llid=5608)(MAP2K6) [580](http://genereg.ornl.gov/gotm/llid_info.php?llid=580)(BARD1)  [anti-apoptosis*(O=3;E=0.23;R=13.04;P=0.00158444516973)](http://genereg.ornl.gov/gotm/node_id_list_new.php?gotree_id=01-03-03-04-04-01-11-01-01)  [3552](http://genereg.ornl.gov/gotm/llid_info.php?llid=3552)(IL1A) [3558](http://genereg.ornl.gov/gotm/llid_info.php?llid=3558)(IL2) [4170](http://genereg.ornl.gov/gotm/llid_info.php?llid=4170)(MCL1)  [induction of apoptosis by extracellular signals*(O=3;E=0.07;R=42.86;P=5.05776482772E-05)](http://genereg.ornl.gov/gotm/node_id_list_new.php?gotree_id=01-03-03-04-04-01-11-02-01-02)  [10922](http://genereg.ornl.gov/gotm/llid_info.php?llid=10922)(FASTK) [1612](http://genereg.ornl.gov/gotm/llid_info.php?llid=1612)(DAPK1) [1616](http://genereg.ornl.gov/gotm/llid_info.php?llid=1616)(DAXX)  [protein ubiquitination*(O=4;E=0.76;R=5.26;P=0.00652142081313)](http://genereg.ornl.gov/gotm/node_id_list_new.php?gotree_id=01-03-03-11-09-03-13-58-02)  [580](http://genereg.ornl.gov/gotm/llid_info.php?llid=580)(BARD1) [6478](http://genereg.ornl.gov/gotm/llid_info.php?llid=6478)(SIAH2) [7187](http://genereg.ornl.gov/gotm/llid_info.php?llid=7187)(TRAF3) [867](http://genereg.ornl.gov/gotm/llid_info.php?llid=867)(CBL)  [nucleotide-excision repair*(O=2;E=0.06;R=33.33;P=0.00155323820243)](http://genereg.ornl.gov/gotm/node_id_list_new.php?gotree_id=01-03-03-11-23-01-08-13)  [2071](http://genereg.ornl.gov/gotm/llid_info.php?llid=2071)(ERCC3) [7507](http://genereg.ornl.gov/gotm/llid_info.php?llid=7507)(XPA)  [protein binding*(O=15;E=6.45;R=2.33;P=0.00047957314251)](http://genereg.ornl.gov/gotm/node_id_list_new.php?gotree_id=02-02-35)  [1612](http://genereg.ornl.gov/gotm/llid_info.php?llid=1612)(DAPK1) [2071](http://genereg.ornl.gov/gotm/llid_info.php?llid=2071)(ERCC3) [3552](http://genereg.ornl.gov/gotm/llid_info.php?llid=3552)(IL1A) [3558](http://genereg.ornl.gov/gotm/llid_info.php?llid=3558)(IL2) [4170](http://genereg.ornl.gov/gotm/llid_info.php?llid=4170)(MCL1) [5154](http://genereg.ornl.gov/gotm/llid_info.php?llid=5154)(PDGFA) [580](http://genereg.ornl.gov/gotm/llid_info.php?llid=580)(BARD1) [5885](http://genereg.ornl.gov/gotm/llid_info.php?llid=5885)(RAD21) [6478](http://genereg.ornl.gov/gotm/llid_info.php?llid=6478)(SIAH2) [7158](http://genereg.ornl.gov/gotm/llid_info.php?llid=7158)(TP53BP1) [7187](http://genereg.ornl.gov/gotm/llid_info.php?llid=7187)(TRAF3) [7507](http://genereg.ornl.gov/gotm/llid_info.php?llid=7507)(XPA) [841](http://genereg.ornl.gov/gotm/llid_info.php?llid=841)(CASP8) [842](http://genereg.ornl.gov/gotm/llid_info.php?llid=842)(CASP9) [867](http://genereg.ornl.gov/gotm/llid_info.php?llid=867)(CBL)  [nucleus*(O=16;E=8.09;R=1.98;P=0.00147434526058)](http://genereg.ornl.gov/gotm/node_id_list_new.php?gotree_id=03-06-02-02-13)  [142](http://genereg.ornl.gov/gotm/llid_info.php?llid=142)(PARP1) [1616](http://genereg.ornl.gov/gotm/llid_info.php?llid=1616)(DAXX) [2071](http://genereg.ornl.gov/gotm/llid_info.php?llid=2071)(ERCC3) [472](http://genereg.ornl.gov/gotm/llid_info.php?llid=472)(ATM) [5395](http://genereg.ornl.gov/gotm/llid_info.php?llid=5395)(PMS2) [5725](http://genereg.ornl.gov/gotm/llid_info.php?llid=5725)(PTBP1) [580](http://genereg.ornl.gov/gotm/llid_info.php?llid=580)(BARD1) [5885](http://genereg.ornl.gov/gotm/llid_info.php?llid=5885)(RAD21) [6117](http://genereg.ornl.gov/gotm/llid_info.php?llid=6117)(RPA1) [6478](http://genereg.ornl.gov/gotm/llid_info.php?llid=6478)(SIAH2) [6773](http://genereg.ornl.gov/gotm/llid_info.php?llid=6773)(STAT2) [7158](http://genereg.ornl.gov/gotm/llid_info.php?llid=7158)(TP53BP1) [7507](http://genereg.ornl.gov/gotm/llid_info.php?llid=7507)(XPA) [8438](http://genereg.ornl.gov/gotm/llid_info.php?llid=8438)(RAD54L) [867](http://genereg.ornl.gov/gotm/llid_info.php?llid=867)(CBL) [892](http://genereg.ornl.gov/gotm/llid_info.php?llid=892)(CCNC) |
| Plastic_5 | [mitotic cell cycle*(O=2;E=0.06;R=33.33;P=0.00152642641634)](http://genereg.ornl.gov/gotm/node_id_list_new.php?gotree_id=01-03-03-03-08)  [4085](http://genereg.ornl.gov/gotm/llid_info.php?llid=4085)(MAD2L1) [8558](http://genereg.ornl.gov/gotm/llid_info.php?llid=8558)(CDK10)  [chromosome*(O=2;E=0.06;R=33.33;P=0.00129695892046)](http://genereg.ornl.gov/gotm/node_id_list_new.php?gotree_id=03-01-14-03)  [4085](http://genereg.ornl.gov/gotm/llid_info.php?llid=4085)(MAD2L1) [5111](http://genereg.ornl.gov/gotm/llid_info.php?llid=5111)(PCNA) |
| Plastic_6 | [cell cycle arrest*(O=2;E=0.11;R=18.18;P=0.0052107339862)](http://genereg.ornl.gov/gotm/node_id_list_new.php?gotree_id=01-03-05-05-05-01)  [4853](http://genereg.ornl.gov/gotm/llid_info.php?llid=4853)(NOTCH2) [5036](http://genereg.ornl.gov/gotm/llid_info.php?llid=5036)(PA2G4)  [nucleus*(O=11;E=4.8;R=2.29;P=0.00154209636493)](http://genereg.ornl.gov/gotm/node_id_list_new.php?gotree_id=03-01-14-28)  [1017](http://genereg.ornl.gov/gotm/llid_info.php?llid=1017)(CDK2) [1063](http://genereg.ornl.gov/gotm/llid_info.php?llid=1063)(CENPF) [1161](http://genereg.ornl.gov/gotm/llid_info.php?llid=1161)(ERCC8) [2189](http://genereg.ornl.gov/gotm/llid_info.php?llid=2189)(FANCG) [4853](http://genereg.ornl.gov/gotm/llid_info.php?llid=4853)(NOTCH2) [5036](http://genereg.ornl.gov/gotm/llid_info.php?llid=5036)(PA2G4) [5245](http://genereg.ornl.gov/gotm/llid_info.php?llid=5245)(PHB) [5928](http://genereg.ornl.gov/gotm/llid_info.php?llid=5928)(RBBP4) [595](http://genereg.ornl.gov/gotm/llid_info.php?llid=595)(CCND1) [8099](http://genereg.ornl.gov/gotm/llid_info.php?llid=8099)(CDK2AP1) [996](http://genereg.ornl.gov/gotm/llid_info.php?llid=996)(CDC27) |
| Plastic_7 | [negative regulation of cellular process*(O=4;E=0.64;R=6.25;P=0.00298413923453)](http://genereg.ornl.gov/gotm/node_id_list_new.php?gotree_id=01-03-05-01)  [332](http://genereg.ornl.gov/gotm/llid_info.php?llid=332)(BIRC5) [4504](http://genereg.ornl.gov/gotm/llid_info.php?llid=4504)(MT3) [5999](http://genereg.ornl.gov/gotm/llid_info.php?llid=5999)(RGS4) [7076](http://genereg.ornl.gov/gotm/llid_info.php?llid=7076)(TIMP1)  [antioxidant activity*(O=2;E=0.05;R=40;P=0.00096196402673)](http://genereg.ornl.gov/gotm/node_id_list_new.php?gotree_id=02-01)  [4504](http://genereg.ornl.gov/gotm/llid_info.php?llid=4504)(MT3) [7296](http://genereg.ornl.gov/gotm/llid_info.php?llid=7296)(TXNRD1)  [extracellular matrix*(O=3;E=0.41;R=7.32;P=0.00714672906786)](http://genereg.ornl.gov/gotm/node_id_list_new.php?gotree_id=03-03)  [4316](http://genereg.ornl.gov/gotm/llid_info.php?llid=4316)(MMP7) [4320](http://genereg.ornl.gov/gotm/llid_info.php?llid=4320)(MMP11) [7076](http://genereg.ornl.gov/gotm/llid_info.php?llid=7076)(TIMP1) |
| SISgel_1 | [cell cycle*(O=4;E=0.7;R=5.71;P=0.00418440122301)](http://genereg.ornl.gov/gotm/node_id_list_new.php?gotree_id=01-03-03-03)  [1026](http://genereg.ornl.gov/gotm/llid_info.php?llid=1026)(CDKN1A) [3553](http://genereg.ornl.gov/gotm/llid_info.php?llid=3553)(IL1B) [5155](http://genereg.ornl.gov/gotm/llid_info.php?llid=5155)(PDGFB) [5597](http://genereg.ornl.gov/gotm/llid_info.php?llid=5597)(MAPK6)  [induction of apoptosis*(O=2;E=0.14;R=14.29;P=0.0086682924147)](http://genereg.ornl.gov/gotm/node_id_list_new.php?gotree_id=01-03-03-04-04-01-11-02-01)  [1026](http://genereg.ornl.gov/gotm/llid_info.php?llid=1026)(CDKN1A) [843](http://genereg.ornl.gov/gotm/llid_info.php?llid=843)(CASP10)  [extracellular matrix*(O=3;E=0.38;R=7.89;P=0.00562858470244)](http://genereg.ornl.gov/gotm/node_id_list_new.php?gotree_id=03-03)  [4320](http://genereg.ornl.gov/gotm/llid_info.php?llid=4320)(MMP11) [4326](http://genereg.ornl.gov/gotm/llid_info.php?llid=4326)(MMP17) [7076](http://genereg.ornl.gov/gotm/llid_info.php?llid=7076)(TIMP1) |
| SISgel_2 | [regulation of cell cycle*(O=4;E=0.43;R=9.3;P=0.000691683530146)](http://genereg.ornl.gov/gotm/node_id_list_new.php?gotree_id=01-03-03-03-09)  [3552](http://genereg.ornl.gov/gotm/llid_info.php?llid=3552)(IL1A) [5154](http://genereg.ornl.gov/gotm/llid_info.php?llid=5154)(PDGFA) [5473](http://genereg.ornl.gov/gotm/llid_info.php?llid=5473)(PPBP) [5595](http://genereg.ornl.gov/gotm/llid_info.php?llid=5595)(MAPK3)  [negative regulation of cell proliferation*(O=3;E=0.14;R=21.43;P=0.000334995148912)](http://genereg.ornl.gov/gotm/node_id_list_new.php?gotree_id=01-03-03-10-11-01)  [3456](http://genereg.ornl.gov/gotm/llid_info.php?llid=3456)(IFNB1) [3552](http://genereg.ornl.gov/gotm/llid_info.php?llid=3552)(IL1A) [3586](http://genereg.ornl.gov/gotm/llid_info.php?llid=3586)(IL10)  [inflammatory response*(O=3;E=0.21;R=14.29;P=0.0010116780193)](http://genereg.ornl.gov/gotm/node_id_list_new.php?gotree_id=01-07-15-01-06-05-06)  [3440](http://genereg.ornl.gov/gotm/llid_info.php?llid=3440)(IFNA2) [3552](http://genereg.ornl.gov/gotm/llid_info.php?llid=3552)(IL1A) [3586](http://genereg.ornl.gov/gotm/llid_info.php?llid=3586)(IL10) |
| SISgel_3 | [induction of apoptosis by extracellular signals*(O=2;E=0.06;R=33.33;P=0.00140280893016)](http://genereg.ornl.gov/gotm/node_id_list_new.php?gotree_id=01-03-03-04-04-01-11-02-01-02)  [10922](http://genereg.ornl.gov/gotm/llid_info.php?llid=10922)(FASTK) [1612](http://genereg.ornl.gov/gotm/llid_info.php?llid=1612)(DAPK1)  [DNA replication*(O=4;E=0.29;R=13.79;P=0.000171553801582)](http://genereg.ornl.gov/gotm/node_id_list_new.php?gotree_id=01-03-03-11-23-01-09)  [5245](http://genereg.ornl.gov/gotm/llid_info.php?llid=5245)(PHB) [6240](http://genereg.ornl.gov/gotm/llid_info.php?llid=6240)(RRM1) [7150](http://genereg.ornl.gov/gotm/llid_info.php?llid=7150)(TOP1) [7153](http://genereg.ornl.gov/gotm/llid_info.php?llid=7153)(TOP2A)  [purine nucleotide biosynthesis*(O=3;E=0.15;R=20;P=0.000399426317)](http://genereg.ornl.gov/gotm/node_id_list_new.php?gotree_id=01-03-03-11-23-07-06-06)  [4522](http://genereg.ornl.gov/gotm/llid_info.php?llid=4522)(MTHFD1) [471](http://genereg.ornl.gov/gotm/llid_info.php?llid=471)(ATIC) [8833](http://genereg.ornl.gov/gotm/llid_info.php?llid=8833)(GMPS  [ligase activity, forming carbon-nitrogen bonds*(O=5;E=0.74;R=6.76;P=0.000719739882372)](http://genereg.ornl.gov/gotm/node_id_list_new.php?gotree_id=02-03-19-04)  [4522](http://genereg.ornl.gov/gotm/llid_info.php?llid=4522)(MTHFD1) [7187](http://genereg.ornl.gov/gotm/llid_info.php?llid=7187)(TRAF3) [7188](http://genereg.ornl.gov/gotm/llid_info.php?llid=7188)(TRAF5) [7726](http://genereg.ornl.gov/gotm/llid_info.php?llid=7726)(TRIM26) [8833](http://genereg.ornl.gov/gotm/llid_info.php?llid=8833)(GMPS)  [cytoskeleton*(O=6;E=1.26;R=4.76;P=0.00105764014731)](http://genereg.ornl.gov/gotm/node_id_list_new.php?gotree_id=03-01-14-07)  [1495](http://genereg.ornl.gov/gotm/llid_info.php?llid=1495)(CTNNA1) [1499](http://genereg.ornl.gov/gotm/llid_info.php?llid=1499)(CTNNB1) [1612](http://genereg.ornl.gov/gotm/llid_info.php?llid=1612)(DAPK1) [3856](http://genereg.ornl.gov/gotm/llid_info.php?llid=3856)(KRT8) [3875](http://genereg.ornl.gov/gotm/llid_info.php?llid=3875)(KRT18) [7283](http://genereg.ornl.gov/gotm/llid_info.php?llid=7283)(TUBG1) |
| SISgel_4 |  |
| SISgel_5 | [negative regulation of cell cycle*(O=3;E=0.07;R=42.86;P=3.94070781832E-05)](http://genereg.ornl.gov/gotm/node_id_list_new.php?gotree_id=01-03-05-01-03-04)  [10301](http://genereg.ornl.gov/gotm/llid_info.php?llid=10301)(DLEU1) [4292](http://genereg.ornl.gov/gotm/llid_info.php?llid=4292)(MLH1) [5378](http://genereg.ornl.gov/gotm/llid_info.php?llid=5378)(PMS1) |
| SISgel_6 | [cell division*(O=4;E=0.26;R=15.38;P=0.000111330424545)](http://genereg.ornl.gov/gotm/node_id_list_new.php?gotree_id=01-03-03-05)  [1017](http://genereg.ornl.gov/gotm/llid_info.php?llid=1017)(CDK2) [4085](http://genereg.ornl.gov/gotm/llid_info.php?llid=4085)(MAD2L1) [4735](http://genereg.ornl.gov/gotm/llid_info.php?llid=4735)(SEPT2) [983](http://genereg.ornl.gov/gotm/llid_info.php?llid=983)(CDC2)  [protein complex assembly*(O=3;E=0.4;R=7.5;P=0.00709797718378)](http://genereg.ornl.gov/gotm/node_id_list_new.php?gotree_id=01-07-10-09-05-01-10)  [6892](http://genereg.ornl.gov/gotm/llid_info.php?llid=6892)(TAPBP) [915](http://genereg.ornl.gov/gotm/llid_info.php?llid=915)(CD3D) [972](http://genereg.ornl.gov/gotm/llid_info.php?llid=972)(CD74) |
