# Supplementary figures and images for: Analysis of the interaction of extracellular matrix and phenotype of bladder cancer cells
Source: BMC Cancer. 2006 Jan 13;6:12. doi: 10.1186/1471-2407-6-12 (PMC1360102; doi:10.1186/1471-2407-6-12)

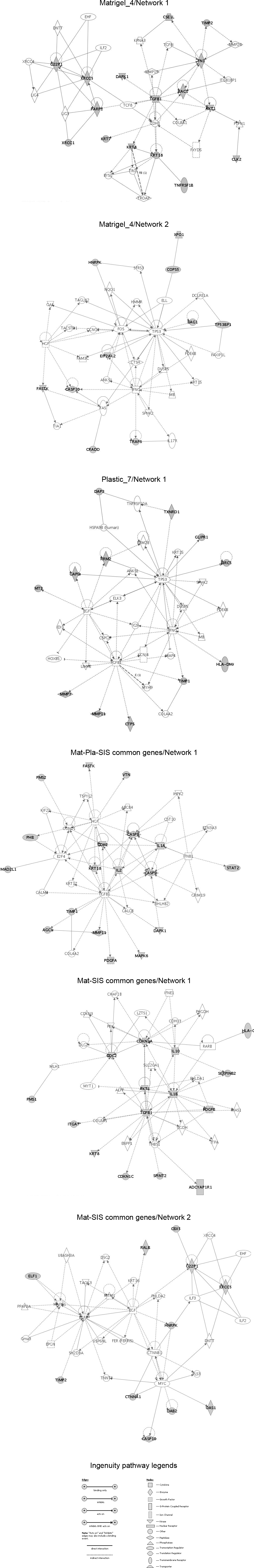

Supplement: Additional File 6 — Figure 2. Gene networks identified by Ingenuity analysis. Pathways for Matrigel, cluster 4, Plastic, cluster 7, common genes between all three matrixes, common genes between Matrigel and SISgel are shown. Focus genes that map to the Global Molecular Network are displayed with bold text. User input genes are emphasized by gray color. For pathway legend see bottom of the figure. [file 1471-2407-6-12-S6.jpeg]
